# Research on supply chain performance based on retailers’ fairness concerns: Wholesale prices versus cost sharing of efforts

**DOI:** 10.1371/journal.pone.0204482

**Published:** 2018-10-24

**Authors:** Xiaoguang Liu, Xifu Wang, Lufeng Dai, Yanfang Pan

**Affiliations:** School of Traffic and Transportation, Beijing Jiaotong University, Beijing, China; Shandong University of Science and Technology, CHINA

## Abstract

With the deepening of the research on supply chain management, scholars have increasingly begun to investigate the impact of fairness on a supply chain, and many conclusions suggest that a simple wholesale price can coordinate a supply chain under specific conditions. However, the corresponding analysis and other optimization mechanisms that affect the situation in which the channel cannot be coordinated are either omitted or given little attention. In this paper, we constructed a dyadic supply chain with a single manufacturer and a single retailer; the manufacturer acts as a selfish leader, and the retailer acts as a follower with fairness concerns and sales efforts. For this setting, we derived the equilibrium strategy solution for a wholesale price contract and cost sharing of effort (CS-E) contract offered by the manufacturer, and the results indicated that both contracts achieved channel coordination with different requirements. Further, the profit of the manufacturer and the sales effort of the retailer under CS-E contracts were never less than those for the wholesale price contract, and there was an interval during which the retailer's profit and utility and supply chain efficiency were better than those under the wholesale price contract. In addition, we described situations in which a CS-E contract is unnecessary. These results should be a useful reference for managerial decisions and organizations.

## Introduction

It is well known that traditional supply chain management (SCM) is based on the assumption that decision makers are completely rational. However, there is now empirical evidence that fairness considerations significantly affect economic behaviour. For example, Britain's largest retailer, Marks & Spencer, benefits by treating its suppliers fairly and thus has established long-term, stable partnerships [1]. In contrast, Langsha Group and Xuzhou Wanji Trading chose to disrupt cooperation with a partner who seized a disproportionate share of the profits [2]. A large number of investigations have confirmed that firms have fairness preferences as individuals do in business [3,4]. This evidence presents sufficient reasons for us to revisit some traditional achievements from the perspective of fairness.

In the past, the coordination in decentralized channels has gained considerable attention from both practitioners and researchers. The primary purpose of coordination is to design a contract to avoid the negative impact of "double marginalization" and improve the overall efficiency of the supply chain. Many coordinating contracts were proposed in the early studies, such as buy-back (BB), quantity discounts (QD), revenue-sharing (RS), two-part tariff (TPT) and so on [5]. However, some laboratory experiments with human subjects indicate that these contracts, which have been shown to be quite effective in theory, perform poorly in practice [6–10]. The main reason is that in the supply chain contract environment, some behavioural factors, such as profit allocation between supply chain members that trigger fairness concerns behaviour, affect the performance of the supply chain [11,12].

Retail pricing is not only an important vehicle to enhance supply chain revenue but also an important means for retailers to pursue the fair allocation of channel profits. Some studies have incorporated fairness concerns into typical supply chain models with price-dependent demand; these studies demonstrate that a wholesale price can be designed to achieve channel coordination in this situation. In addition to price-only strategy, in most situations, sales efforts are also important in influencing demand. In practice, retailers can attempt to attract more consumers and spur marketing demand using various forms of sales efforts, such as merchandising, commercial advertising, purchase guiding or other personalized services [13, 14]. In addition, it is vital to make the optimal sales effort decisions to improve the competitiveness of the channel and bring more investment opportunities [15]. Although sales efforts are significant in the development of the supply chain, the corresponding cost burden falls upon the retailers, rendering it challenging for them to undertake the optimal initiatives. More importantly, retailers may refuse to endorse the original profit allocation and show a stronger concern about fairness when the gain was based on their sales performance [16].

To stimulate the sales activities of the downstream retailers, the core enterprises of the supply chain often provide the necessary support for retailers. Their goal is to increase product sales in the terminal market and enhance profits throughout the supply chain. Cost sharing of effort (CS-E) is an effective incentive for improving the performance of supply chains in practice. For example, Dongfanghong, one of the largest green onion packers in China, provides 60 percent of the pesticides its contract farmers need [17]. Similarly, Intel paid approximately $1.5 billion to its retailers to promote its products, which equates to 60% of its retailers' promotion costs [18]. Nagler (2006) conducted a large-scale empirical study on 2,286 brands and determined that over 60% of the brands adopted vertical cooperative advertising [19]. The majority of studies have also theoretically confirmed that supply-chain performance improves under the CS-E, which is significantly better than the wholesale price contract [20–22]. Therefore, how should CS-E contracts be designed when considering retailers' concerns about fairness? Further, does a CS-E contract remain necessary if the supply chain can achieve coordination through the wholesale price contract when fairness exists?

Considering the above motivations, we constructed a dyadic supply chain with a single manufacturer and a single retailer; the manufacturer acts as a selfish leader, and the retailer acts as a follower with fairness concerns. The market demand is sensitive to retail prices and sales efforts. We investigated the considered model with two types of contracts: wholesale price and CS-E contracts. With the former, the manufacturer only sets the wholesale price during the game. With the latter, in addition to setting wholesale prices, manufacturers must also decide how to share the effort costs incurred by retailers. This paper seeks to answer the following questions: a) how do the fairness concerns of the retailer influence the optimal strategy in a decentralized channel under two different contracts? b) What is the cross effect between fairness concerns and sales efforts in conditions of channel coordination? c) Compared with the wholesale price contract, can the cost-sharing contract improve channel performance and achieve coordination? d) Which contract should we address in which conditions from the perspectives of manufacturers, retailers and the entire channel?

The primary contributions of this paper are as follows: First, we extend the supply chain systems considered in the literature to include fairness concerns and price/effort-dependent demand. Specifically, manufacturers and retailers each have at least one decision variable in a considered model, which renders our objective problems much more complex. By deriving equilibrium, we determined that sales efforts under the wholesale price contract do not significantly affect the manufacturer's optimal decision. Second, both contracts can coordinate the supply chain but under different conditions; i.e., the CS-E contract requires less stringent conditions than a solely wholesale price to achieve coordination when the cost coefficient is in a low position and vice versa. Finally, this paper presents a comprehensive comparison of each optimal strategy and corresponding performance between the two contracts. We suggest that sales efforts and manufacturers' payoffs are always higher under CS-E and that there is also an interval during which the CS-E absolutely performs better than the wholesale price-only for all aspects of channel performance.

The remainder of the paper is organized as follows: Section 2 describes the essential features of the research setting. In Sections 3 and 4, we analyse the fairness model in detail and derive the equilibria for simultaneous wholesale price contracts and CS-E contracts. We compare and analyse the two contracts in Section 5, and Section 6 summarizes our conclusions and presents ideas for future research.

## Literature review

In this paper, we review literature in three streams. The first section discusses works that incorporated fairness into supply chain models. The second section discusses the studies on channel coordination and fairness concerns. The third section discusses the cost-sharing contract as applied to channel coordination.

The first stream is related to the literature on supply chain models with fairness concerns. There are by now two categories among the relevant works incorporating fairness concerns into supply chain models. One category concerns distributional fairness between upstream and downstream channels; the other is about peer-induced fairness concerns, which characterizes the fairness preferences of multiple agents who are in similar circumstances. Our work is related to the former by examining distributional fairness between retailer and manufacturer. Fehr and Schmidt (1999) [23] proposed the so-called 'inequity aversion' model in which a player has a disutility of receiving a payoff that is most commonly employed to describe distributional fairness. Based on Fehr and Schmidt (1999) [23], Cui (2007) [24] and Ozgun et al. (2010) [25] investigated the performance of the fair channel. However, these early studies only considered demand to be sensitive to retail price and did not include non-price factors. Wei and Lin (2014) explored the effects of fairness concerns on effort strategies with non-linear stochastic demand [26]. Ge (2015) studied effort decisions in the retailer-dominated supply chain considering suppliers’ fairness. In these studies, rather than a decision variable, the retail price was exogenous or not considered [27]. Li and Li (2016) considered a dual-channel supply chain in which a manufacturer produces a single product and sells the product through a direct channel and also through a traditional retail channel in which the retailer has fairness concerns and provides both retail price and value-added services to consumers. In their models, the authors determined that the optimal value-added services set by retailers are always at a fixed value independent of the rival’s decision [28]. Li Q et al. (2018) studied how fairness concerns influence price and sales effort decisions in a single channel [29]. They adopted a simplified 'inequity aversion' model that omitted the advantageous inequality of players. To make the research more closely reflect conditions of reality, our model considers market demand, which is affected by both sales efforts and retail prices, without simplifying the utility function of fairness concerns.

The second stream is those studies on channel coordination and fairness concerns. Coordination is an important issue in supply chain management. To resolve this problem, various coordinating contracts with fairness concerns have been proposed in different supply chain structures pioneered by Cui et al. [24], who suggested that a simple wholesale price above the marginal cost can be used by the manufacturer that is in the leadership position to coordinate this channel both in terms of achieving the maximum channel profit and obtaining the maximum channel utility if retailers have a strong sense of fairness. The results obtained by Cui et al. [24] were extended by Ozgun et al. [25] to other nonlinear demand functions that are frequently used in this research area. The exponential demand function requires less stringent conditions than the linear demand function to achieve coordination. Du, Nie, Chu, and Yu (2014) suggested that the channel can also be coordinated with a simple wholesale price when the supplier and the retailer both have preferences for reciprocity [30]. Katok and Pavlov (2014) observed that a linear pricing contract can nevertheless maximize the channel profit when there is information asymmetry between channel members [31]. In addition to the wholesale price contract, some works also discussed the improvement effect of other traditional contracts on supply chain fairness concerns. Pavlov and Katok (2015) suggested that the optimal contract to enhance channel efficiency can be implemented with a minimum-order-quantity contract but not with a two-part tariff when retailers’ preferences are private information [32]. Nie and Du (2016) considered peer-induced fairness and distributional fairness simultaneously and proposed a coordination mechanism that combined quantity discount contracts with fixed fees [2]. Bi, He, and Luo et al. (2013) demonstrated that a sales-rebate contract regarding fairness broadens the voluntary cooperation prospects and increases the overall profit of the supply chain [33]. Some other coordinating contracts with fairness concerns have also been fully characterized in different contexts, such as revenue-sharing contracts (Li Q et al [29]; Wang X et al. [34]; Pu X J et al. [35]) and buy-back contracts (Wei G et al [36,37]). Although these articles considered more complex issues, the majority of them also simplified the functions that characterize fairness concerns.

The final stream of literature related to our article concerns the cost-sharing contract on sales efforts as applied to channel coordination. Contracts of cost sharing in supply chains have been studied extensively, and a substantial number of studies considered supply chains’ coordination with sales efforts in different situations. For example, Wang and Gerchak (2001) studied a model in which the retailer's shelf space was treated as the retailer's inventory-holding effort. They developed a contract that allowed the supplier to share the retailer's effort costs to coordinate the supply chain [38]. Sana S S (2013) constructed a game model considering production-inventory with uncertain promotional efforts and suggested that sharing a contract on promotional efforts provided by the manufacturer aligned the incentives of the members of the chain [39]. Tsao and Sheen (2013) examined retailers’ promotion and replenishment decisions under retailer competition and promotional effort conditions with the sales learning curves. The results indicated that keeping the fractions of promotion cost sharing within an appropriate range significantly increases profits for all parties [40]. Ghosh and Shah (2015) demonstrated that an appropriate service-cost sharing contract proposed by a manufacturer opening online channels can be designed to effectively stimulate the service level of the off-line retailer while free riding occurs [41]. However, the majority of these previous studies focused on cost sharing of sales efforts based on an assumption of perfect rationality in supply chains. This paper is most closely related to Zhou et al (2016). [42], who surveyed the role of co-op advertising (CA) contracts in coordinating a low-carbon supply chain considering the retailer’s fairness concerns. However, our paper differs from theirs in several respects. First, the demand function in their models was only sensitive to members’ green efforts whereas we consider the cross-effect of retail price and sales effort on consumer demand. Second, the cost-sharing proportion and wholesale price offered by the manufacturer are both exogenous to their work whereas they serve as the decision variables for the manufacturer in our models. Further, the results of Zhou et al. [42] demonstrated that a unilateral cost-sharing contract cannot coordinate the channel, which is not consistent with this study.

## Model description

This paper considers the standard dyadic channel comprising a single manufacturer and a single retailer. The manufacturer and retailer play a classical Stackelberg game, and the manufacturer produces a product at unit manufacturing cost *c* and wholesales the product to the retailer at wholesale price *w*. The retailer, in turn, retails it to customers at retail price *p* over a single selling season. We assume that the retailer can influence the demand by exerting marketing effort at a cost that equals $\frac{K}{2}e^{2}$, where *e* is the effort level and *K* is the marketing cost coefficient. Following Gurnani and Erkoc (2008) [43], we assume that the demand information is symmetrically known to both members and that the demand function is a normalized demand, i.e., *D*(*p*,*e*) = *a* − *p* + *le*, where *a* is the base market size and it is common information with *a* > *p*. *l* measures the sales effort elasticity. The profits of the manufacturer and the retailer may be respectively expressed as

$$\Pi^{r}(p,e)=(p-w)D(p,e)-\frac{K}{2}e^{2},$$ (1)

$$\Pi^{m}(w)=(w-c)D(p,e).$$ (2)

Thus, the profit of the whole supply chain can be described as

$$\Pi^{c}(p,e)=(p-c)(a-p+le)-\frac{K}{2}e^{2}.$$ (3)

When both parties act as an integrated system in (3), it is possible to obtain the centralized channel profit of $\frac{K{\left(a-c\right)}^{2}}{2(2K-l^{2})}$, where 2*K*>*l*^2^ with the optimal retail price $p_{c}^{*}=\arg\;\max\Pi^{c}(p,e)=\frac{l^{2}(a-c)}{2(2K-l^{2})}+\frac{a+c}{2}$ and sales effort $e_{c}^{*}=\arg\;\max\Pi^{c}(p,e)=\frac{l(a-c)}{2K-l^{2}}$. It is well known that a supply chain with a traditional decentralized structure fails to reach the upper bound of profit $\frac{K{\left(a-c\right)}^{2}}{2(2K-l^{2})}$ for selfish members.

Given that leaders have more power to share the profit of the entire supply chain, we assume that the retailer has a fairness concern regarding the distribution of profits and that the manufacturer focuses on its own profits. Retailers with fairness concerns will maximize their utility rather than their profits in the decision-making process. According to Fehr and Schmidt (1999) [23], retailers with fairness concerns will maximize their utility rather than their profits in the decision-making process. Thus, the retailer utility can be represented as

$$U^{r}(p,e)=\Pi^{r}(p,e)+f^{r}(p,e).$$ (4)

Here, *Π*^*r*^(*p*,*e*) denotes the monetary profit of the retailer, *f*^*r*^(*p*,*e*) represents the retailer's disutility due to unfairness or inequity, and *f*^*r*^(*p*,*e*)≤0. With reference to the model proposed by Cui (2007), the expression *f*^*r*^(*p*,*e*) can be written as Eq (5), and we provide a brief introduction, considering that many extant studies have described the model in detail.

$$f^{r}(p,e)=-\alpha {\left(\gamma \Pi^{m}(w)-\Pi^{r}(p,e)\right)}^{+}-\beta {\left(\Pi^{r}(p,e)-\gamma \Pi^{m}(w)\right)}^{+},$$ (5)

Three exogenous parameters (*α*,*β*,*γ*) in *f*^*r*^(*p*,*e*) characterize behaviour related to fairness concerns where 0<*β*<1, *α*≥*β* and *γ*>0. *γΠ*^*m*^(*w*) represents a standard of equitable distribution that depends on the monetary payoff of the manufacturer from the retailer's perspective. If the retailer's monetary payoff *Π*^*r*^(*p*,*e*) is lower than the standards *γΠ*^*m*^(*w*), then the retailer will experience disadvantageous disutility with −*α*(*γΠ*^*m*^(*w*)−*Π*^*r*^(*p*,*e*,)). Otherwise, the retailer experiences advantageous disutility −*β*(*Π*^*r*^(*p*,*e*)−*γΠ*^*m*^(*w*)). We denote the parameters *α*,*β* as fairness concern coefficients of the retailer, where *α* denotes the jealous concern coefficient and *β* denotes the guilty concern coefficient; a larger *α*,*β* indicates a stronger sense of fairness, and vice versa.

## Wholesale price contracts

Disutility *f*^*r*^(*p*,*e*) prevents the utility function of the retailer from always being differentiated. Apparently, the retailer can ensure that its profits are either more or less than the fair standards for a wholesale price, which are determined by the manufacturer, and the optimal decision of the retailer will depend on which strategy results in the optimum utility. This process can be reduced to (6):

$$U^{r}(p^{*},e^{*})=\max\left\{\underset{\Pi^{r}\leq \gamma \Pi^{s}}{\max}U^{r}(p,e),\;\underset{\Pi^{r}\geq \gamma \Pi^{s}}{\max}U_{}^{r}(p,e)\right\}.$$ (6)

The sub-problem of Eq (6) in the region *Π*^*r*^(*p*,*e*)≥*γΠ*^*m*^(*p*,*e*) can be expressed as

$$\begin{array}{l} \underset{p,e}{\max}\;U^{r}(p,e)=\Pi^{r}(p,e)-\beta \cdot [\Pi^{r}(p,e)-\gamma \Pi^{m}(p,e)]\\ s.t.\;\Pi^{r}(p,e)\geq \gamma \Pi^{m}(p,e) \end{array}.$$ (7)

Note that the Hessian matrix of (7) is a negative definite for all *p*,*e* if *K*,*l* satisfy the condition 2*K*−*l*^2^>0. (*p*_1_,*e*_1_) and (*p*_2_,*e*_2_) are the regional equilibrium solutions and the boundary equilibrium solution under the constraints *Π*^*r*^(*p*,*e*)≥*γΠ*^*m*^(*p*,*e*), respectively. Then, the local optimal response strategy of the retailer in (7) can be expressed as

$$(p,e)=\left\{ \begin{array}{l} (p_{1},e_{1})\;\mathrm{if}\;w\leq {\tilde{w}}_{1}\\ (p_{2},e_{2})\;\mathrm{otherwise} \end{array}\right.$$ (8)

where

$$\begin{array}{l} {\tilde{w}}_{1}=\frac{(1-\beta )a+(2-\beta )\gamma c}{1-\beta -\beta \gamma +2\gamma }\\ (p_{1},e_{1})=(\frac{[(1-\beta -\beta \gamma )w(K-l^{2})+\beta \gamma c]+(1-\beta )aK}{\left(1-\beta )(2K-l^{2}\right)},\frac{\left(1-\beta )(a-w)+\beta \gamma (w-c\right)}{\left(1-\beta )(2K-l^{2}\right)}l),\\ (p_{2},e_{2})=(\frac{l^{2}(a-c)}{2(2K-l^{2})}+\frac{a+c}{2},\frac{l(a-c)}{2K-l^{2}}). \end{array}$$

Note that (*p*_2_,*e*_2_) is the equilibrium solution under constraint *Π*^*r*^(*p*,*e*) = *γΠ*^*m*^(*p*,*e*); obviously, the profit of the supply chain can be expressed as $\Pi^{c}(p,e)=(1+\frac{1}{\gamma })\cdot \Pi^{r}(p,e)$. Thus, the local optimal strategy (*p*_2_,*e*_2_) is equal to $\left(p_{c}^{*},e_{c}^{*}\right)$, which is the optimal strategy in a centralized channel, as previously mentioned.

Similarly, another sub-problem of (6) in the region *Π*^*r*^(*p*,*e*)<*γΠ*^*m*^(*p*,*e*) is the following:

$$\begin{array}{l} \underset{p,e}{\max}\;U^{r}(p,e)=\Pi^{r}(p,e)-\alpha \cdot [\gamma \Pi^{m}(p,e)-\Pi^{r}(p,e)]\\ s.t.\;\Pi^{r}(p,e)<\gamma \Pi^{m}(p,e). \end{array}$$ (9)

Here, we define (*p*_3_,*e*_3_) as the regional equilibrium solution derived under *Π*^*r*^(*p*,*e*)<*γΠ*^*m*^(*p*,*e*), and it is simple to find the equilibrium solution on the boundary, which remains (*p*_2_,*e*_2_) in this case. Then, we have the retailer's local optimal strategy (10):

$$(p,e)=\left\{ \begin{array}{l} (p_{3},e_{3})\;\mathrm{if}\;w>{\tilde{w}}_{2}\\ (p_{2},e_{2})\;\mathrm{otherwise} \end{array}\right.$$ (10)

where

$$\begin{array}{l} {\tilde{w}}_{2}=\frac{(1+\alpha )a+(2+\alpha )\gamma c}{1+\alpha +\alpha \gamma +2\gamma }\\ \left(p_{3},e_{3})=(\frac{[(1+\alpha +\alpha \gamma )w(K-l^{2})-\alpha \gamma c]+(1+\alpha )aK}{\left(1+\alpha )(2K-l^{2}\right)},\frac{\left(1+\alpha )(a-w)-\alpha \gamma (w-c\right)}{\left(1+\alpha )(2K-l^{2}\right)}l\right) \end{array}$$

Then, the global optimal solution according to (8) and (10) is shown in Proposition 1.

**Proposition 1.** If the retailer is the only supply chain member with fairness concerns, his global optimal strategy (p*(*w*),e*(*w*)) for wholesale price *w*, which is determined by the manufacturer, is shown below:

$$(p^{*}(w),e^{*}(w))=\left\{ \begin{array}{l} (p_{1},e_{1})\;\mathrm{if}\;w\leq {\tilde{w}}_{1}\\ (p_{2},e_{2})\;\mathrm{if}\;{\tilde{w}}_{1}<w\leq {\tilde{w}}_{2}\\ (p_{3},e_{3})\;\mathrm{if}\;w>{\tilde{w}}_{2} \end{array}\right.$$ (11)

The above results show that this can result in the inequitable distribution of supply chain profit from the perspective of the retailer when the wholesale price is set either too high or too low whereas the retailer will have a neutral fairness response if the wholesale price is within a middle interval, which will result in maximum profits for the entire channel. This seems to be a good solution for coordinating if the manufacturers are willing to accept profit distribution such that $\Pi^{m}(p,e)=\frac{1}{\gamma }\Pi^{r}(p,e)$.

On the basis of (11), the manufacturer obtained the local maximum profit $\Pi_{i}^{m}$, corresponding to each local optimal strategy (*p*_*i*_,*e*_*i*_), *i* = 1,2,3 of the retailer and then determined her global optimal strategy comparing each $\Pi_{i}^{m}$. Hence, the optimization problem for the manufacturers can be expressed as follows:

$$\Pi^{m}(w^{*})=\max\left\{\underset{w\leq {\tilde{w}}_{1}}{\max}\Pi_{1}^{m}(w),\;\underset{{\tilde{w}}_{1}<w\leq {\tilde{w}}_{2}}{\max}\Pi_{2}^{m}(w),\;\underset{w>{\tilde{w}}_{2}}{\max}\Pi_{3}^{m}(w)\right\}$$ (12)

The unconstrained maximizers of $\Pi_{1}^{m}(w)$, $\Pi_{2}^{m}(w)$ and $\Pi_{3}^{m}(w)$ are $w_{1}^{*}=\frac{(1-\beta )(a+c)-2\beta \gamma c}{2(1-\beta -\beta \gamma )}$, $w_{2}^{*}=\frac{a-c}{2(1+\gamma )}+c$ and $w_{3}^{*}=\frac{(1+\alpha )(a+c)+2\alpha \gamma c}{2(1+\alpha +\alpha \gamma )}$, respectively. Note that each $w_{i}^{*}$ should satisfy the constraints consisting of ${\tilde{w}}_{1}$, ${\tilde{w}}_{2}$. We provide the conclusions in Proposition 2.

**Proposition 2.** The manufacturer's optimal strategy under a wholesale price contract when the retailer cares about fairness is shown in Table 1.

**Proof.** See Appendix A.

**Table 1:** 

Feasible region	w*	*Π*^*m*^(w*)
**$\beta \leq \frac{1-2\gamma }{1+\gamma }\;and\;\alpha \geq \beta$**	**$w_{1}^{*}$**	**$\frac{K(1-\beta ){\left(a-c\right)}^{2}}{4(1-\beta -\beta \gamma )(2K-l^{2})}$**
**$\frac{1-2\gamma }{1+\gamma }<\beta <\frac{1}{1+\gamma }\;and\;\beta <\alpha \leq \bar{\alpha }$**	**$w_{3}^{*}$**	**$\frac{K(1+\alpha ){\left(a-c\right)}^{2}}{4(1+\alpha +\alpha \gamma )(2K-l^{2})}$**
**$\frac{1-2\gamma }{1+\gamma }<\beta <\frac{1}{1+\gamma }\;and\;\alpha \geq Max\left\{\bar{\alpha },\beta \right\}$**	**${\tilde{w}}_{1}$**	**$\frac{2K\gamma (1-\beta ){\left(a-c\right)}^{2}}{{\left(1-\beta -\beta \gamma +2\gamma \right)}^{2}(2K-l^{2})}$**
**$\beta <\frac{1}{1+\gamma }\;and\;\alpha <Max\left\{\frac{\gamma -1}{1+\gamma },\beta \right\}$**	**${\tilde{w}}_{2}$**	**$\frac{K(1+\alpha ){\left(a-c\right)}^{2}}{4(1+\alpha +\alpha \gamma )(2K-l^{2})}$**
**$\frac{1}{1+\gamma }\leq \beta <1\;and\;\alpha \geq Max\left\{\frac{\gamma -1}{1+\gamma },\beta \right\}$**	**$w_{2}^{*}$**	**$\frac{K{\left(a-c\right)}^{2}}{2(1+\gamma )(2K-l^{2})}$**

Note $\bar{\alpha }=\arg(\Pi_{1}^{m}({\tilde{w}}_{1})=\Pi_{3}^{m}(w_{3}^{*}))=\frac{{\left(1-\beta -\beta \gamma -2\gamma \right)}^{2}-8\beta \gamma^{2}}{8\gamma^{2}-{\left(1-\beta -\beta \gamma -2\gamma \right)}^{2}}$.

According to Table 1, the manufacturer and retailer can simultaneously ensure that the channel achieves both maximum profit and maximum utility $\frac{K{\left(a-c\right)}^{2}}{2(2K-l^{2})}$ when *α*,*β* satisfies the range $\left\{\beta \geq \frac{1}{1+\gamma }\right\}\cap \left\{\alpha \geq Max\left\{\frac{\gamma -1}{1+\gamma },\beta \right\}\right\}$. This finding shows that a "sensitive" retailer with a strong sense of guilt and jealousy should be more motivated to promote supply chain coordination under a wholesale price contract.

Specifically, the manufacturer's optimal strategy for the wholesale price and the corresponding feasible region are consistent with that of Cui et al. (2007), which ignores sales efforts. However, one difference is that the members profit when the retailers' sales efforts are $\frac{2K}{\left(2K-l^{2}\right)}$ times the situation in which the retailer does not exert any sales effort. This implies that exerting sales effort, which increases the profits of both members at the same proportion according to $\frac{2K}{\left(2K-l^{2}\right)}>1$ (2*K*−*l*^2^>0), will not impact either the strategy of selfish manufacturers or the distribution of the channel's profits.

## Cost sharing of effort (CS-E)

In the CS-E model, the manufacturer shares the cost that the retailer incurs for the sales effort. We denote this contract as (*w*,*θ*), where *w* is the unit wholesale price and 0≤*θ*≤1 is the proportion of the sales cost that the manufacturer pays while the retailer assumes the remaining 1−*θ* portion of the cost for the sales effort. The profits of the retailer and the manufacturer are $\Pi^{r}(p,e)=(p-w)D(p,e)-(1-\theta )\frac{K}{2}e^{2}$ and $\Pi^{m}(w)=(w-c)D(p,e)-\frac{K}{2}\theta e^{2}$.

Thus, the retailer's decision under the cost-sharing contract can be expressed as follows:

$$U^{r}(p^{*}(w,\theta ),e^{*}(w,\theta ))=\max\left\{\underset{\Pi^{r}\leq \gamma \Pi^{s}}{\max}U^{r}(p,e),\;\underset{\Pi^{r}\geq \gamma \Pi^{s}}{\max}U^{r}(p,e)\right\}$$ (13)

(*p*_*θ*,*i*_,*e*_*θ*,*i*_) is the retailer's local equilibrium strategy under CS-E contracts, *i* = 1,2,3. The local equilibrium strategy for the case *Π*^*r*^(*p*,*e*)>*γΠ*^*m*^(*p*,*e*) is given by

$\begin{array}{l} (p_{\theta ,1},e_{\theta ,1})=\\ \left(\frac{\left(1-\beta )(a-w)+\beta \gamma (w-c\right)}{2(2Kt_{\beta }-(1-\beta )l^{2})}l^{2}+\frac{\left(1-\beta -\beta \gamma )w+[(1-\beta )a+\beta \gamma c\right]}{2(1-\beta )},\;\frac{\left(1-\beta )(a-w)+\beta \gamma (w-c\right)}{2Kt_{\beta }-(1-\beta )l^{2}}l\right) \end{array}$, where *t*_*β*_ = (1−*β*)−(1−*β*−*βγ*)*θ*.

The local equilibrium strategy when *Π*^*r*^(*p*,*e*)<*γΠ*^*m*^(*p*,*e*) is

$\begin{array}{l} (p_{\theta ,3},e_{\theta ,3})=\\ \left(\frac{\left(1-\beta )(a-w)+\beta \gamma (w-c\right)}{2(2Kt_{\alpha }-(1-\beta )l^{2})}l^{2}+\frac{\left(1-\beta -\beta \gamma )w+[(1-\beta )a+\beta \gamma c\right]}{2(1-\beta )},\;\frac{\left(1-\beta )(a-w)+\beta \gamma (w-c\right)}{2Kt_{\alpha }-(1-\beta )l^{2}}l\right) \end{array}$, where *t*_*α*_ = (1+*α*)−(1+*α*+*αγ*)*θ*.

And the local equilibrium strategy on the boundary *Π*^*r*^(*p*,*e*) = *γΠ*^*m*^(*p*,*e*) is $\left(p_{\theta ,2},e_{\theta ,2})=(\frac{l^{2}(a-c)}{2(2K-l^{2})}+\frac{a+c}{2},\frac{l(a-c)}{2K-l^{2}}\right)$. Depending on the local strategy of the retailer, a comprehensive conclusion may be obtained.

**Proposition 3.** For the manufacturer's decision (*w*,*θ*), the retailer's optimal strategy (p*(*w*,*θ*),e*(*w*,*θ*)) with a fairness concern is shown below:

$$(p^{*}(w,\theta ),e^{*}(w,\theta ))=\left\{ \begin{array}{l} \left(p_{\theta ,1},e_{\theta ,1})\;if\;w(\theta )\leq {\tilde{w}}_{\theta ,1}(\theta \right)\\ \left(p_{\theta ,2},e_{\theta ,2})\;if\;{\tilde{w}}_{\theta ,1}(\theta )<w(\theta )\leq {\tilde{w}}_{\theta ,2}(\theta \right)\\ \left(p_{\theta ,3},e_{\theta ,3})\;if\;w(\theta )>{\tilde{w}}_{\theta ,2}(\theta \right) \end{array}\right.$$ (14)

where

$$\begin{array}{l} {\tilde{w}}_{\theta ,1}(\theta )=\frac{[a-a\beta -\beta \gamma c+2\gamma c][2Kt_{\beta }-(1-\beta )l^{2}]t_{\beta }+(a-a\beta -\beta \gamma c)(1-\beta )\theta \gamma l^{2}}{(1-\beta -\beta \gamma +2\gamma )[2Kt_{\beta }-(1-\beta )l^{2}]t_{\beta }+(1-\beta )(1-\beta -\beta \gamma )\theta \gamma l^{2}}\\ {\tilde{w}}_{\theta ,2}(\theta )=\frac{[a+a\alpha +\alpha \gamma c+2\gamma c][2Kt_{\alpha }-(1+\alpha )l^{2}]t_{\alpha }+(a+a\alpha +\alpha \gamma c)(1+\alpha )\theta \gamma l^{2}}{(1+\alpha +\alpha \gamma +2\gamma )[2Kt_{\alpha }-(1+\alpha )l^{2}]t_{\alpha }+(1+\alpha )(1+\alpha +\alpha \gamma )\theta \gamma l^{2}}. \end{array}$$

Thus, the Hessian matrix of the retailer's utility is a negative definite for (*p*_*θ*,1_,*e*_*θ*,1_) if 2*Kt*_*β*_−(1−*β*)*l*^2^>0. Similarly, we can deduce the other constraint 2*Kt*_*α*_−(1+*α*)*l*^2^>0 if the retailer chooses the strategy (*p*_*θ*,3_,*e*_*θ*,3_), and the constraint 2*K*(1−*θ*)−*l*^2^>0 should hold if (*p*_*θ*,2_,*e*_*θ*,2_). Thus, the above conclusions can be integrated and transformed into the constraint conditions for *θ*, as shown in (15):

$$\left\{ \begin{array}{l} \theta \in [0,{\hat{\theta }}_{1}]\;\mathrm{if}\;w(\theta )\leq {\tilde{w}}_{\theta ,1}(\theta ),\beta <\frac{1}{1+\gamma }\;\mathrm{and}\;2K>l^{2}\\ \theta \in [{\hat{\theta }}_{1},1)\;\mathrm{if}\;w(\theta )\leq {\tilde{w}}_{\theta ,1}(\theta ),\beta \geq \frac{1}{1+\gamma }\;\mathrm{and}\;2K\leq l^{2}\\ \theta \in [0,{\hat{\theta }}_{2}]\;\mathrm{if}\;{\tilde{w}}_{\theta ,1}(\theta )<w(\theta )\leq {\tilde{w}}_{\theta ,2}(\theta )\;\mathrm{and}\;2K>l^{2}\\ \theta \in [0,{\hat{\theta }}_{3}]\;\mathrm{if}\;w(\theta )>{\tilde{w}}_{\theta ,2}(\theta )\;\mathrm{and}\;2K>l^{2} \end{array}\right.$$ (15)

where

$${\hat{\theta }}_{1}=\frac{\left(1-\beta )(2K-l^{2}\right)}{2K(1-\beta -\beta \gamma )},\;{\hat{\theta }}_{2}=\frac{2K-l^{2}}{2K},\;\mathrm{and}\;{\hat{\theta }}_{3}=\frac{\left(1+\alpha )(2K-l^{2}\right)}{2K(1+\alpha +\alpha \gamma )}.$$

$\Pi_{\theta ,i}^{m}$ is the profit of the manufacturer that corresponds to the local strategy (*p*_*θ*,*i*_,*e*_*θ*,*i*_) of the retailer and, in combination with the relevant constraint in (15), allows the global optimal problem of the manufacturer to be given as follows:

$$\Pi^{m}(w^{*},\theta^{*})=\max\left\{\underset{w\leq {\tilde{w}}_{\theta ,1}(\theta )}{\underset{0\leq \theta <{\hat{\theta }}_{1}\;\mathrm{or}\;{\hat{\theta }}_{1}<\theta \leq 1}{\max}}\Pi_{\theta ,1}^{m}(w,\theta ),\underset{{\tilde{w}}_{\theta ,1}(\theta )<w\leq {\tilde{w}}_{\theta ,2}(\theta )}{\underset{0\leq \theta <{\hat{\theta }}_{2}}{\max}}\Pi_{\theta ,2}^{m}(w,\theta ),\underset{w>{\tilde{w}}_{\theta ,2}(\theta )}{\underset{0\leq \theta <{\hat{\theta }}_{3}}{\max}}\Pi_{\theta ,3}^{m}(w,\theta )\right\}.$$ (16)

$\left(w_{\theta ,i}^{*},\theta_{i}^{*}\right)$ is the equilibrium solution of $\Pi_{\theta ,i}^{m}$ without any constraints; then,

$$\begin{array}{l} w_{\theta ,1}^{*}=\frac{\left(1-\beta )[(8K-3l^{2})a+2(4K-3l^{2})c\right]}{\left(1-\beta -\beta \gamma )(16K-9l^{2}\right)}-\frac{\beta \gamma c}{\left(1-\beta -\beta \gamma \right)},\theta_{1}^{*}=\frac{\left(1-\beta \right)}{3(1-\beta -\beta \gamma )},\\ w_{\theta ,2}^{*}=\frac{\left[2K-l^{2}(1-\theta -\theta \gamma )](a-c\right)}{2(2K-l^{2})(1+\gamma )}+c,\theta_{2}^{*}=\forall [0,\frac{2K-l^{2}}{2K})\;\mathrm{and}\\ w_{\theta ,3}^{*}=\frac{\left(1+\alpha )[(8K-3l^{2})a+2(4K-3l^{2})c\right]}{\left(1+\alpha +\alpha \gamma )(16K-9l^{2}\right)}+\frac{\alpha \gamma c}{\left(1+\alpha +\alpha \gamma \right)},\theta_{3}^{*}=\frac{\left(1+\alpha \right)}{3(1+\alpha +\alpha \gamma )}. \end{array}$$

Here, note that the expression $\theta_{1}^{*}$ requires that $\beta <\frac{1}{1+\gamma }$; thus, $\theta_{1}^{*}\in [0,{\hat{\theta }}_{1}]$.

The manufacturer's equilibrium solution will be on one of its boundary constraints when $\left(w_{\theta ,i}^{*},\theta_{i}^{*}\right)$ cannot satisfy the constraints of $\Pi_{\theta ,i}^{m}$ in (16). $\left({\bar{w}}_{\theta ,j},{\bar{\theta }}_{j}\right)$, *j* = 1,2 is the boundary optimal solution of the manufacturer that corresponds to the boundary ${\tilde{w}}_{\theta ,j}(\theta )$; then, the manufacturer's optimal strategy, $\left({\bar{w}}_{\theta ,j},{\bar{\theta }}_{j}\right)$ in this case, can be expressed as

$$\begin{array}{l} ({\bar{w}}_{\theta ,1},{\bar{\theta }}_{1})=({\tilde{w}}_{\theta ,1}({\bar{\theta }}_{1}),\frac{\left(1-\beta \right)}{4K\xi_{\beta }(\xi_{\beta }+4\gamma )}[(4K-l^{2})\xi_{\beta }+(8K-3l^{2})\gamma -\sqrt{\Delta_{1}}]),\\ ({\bar{w}}_{\theta ,2},{\bar{\theta }}_{2})=({\tilde{w}}_{\theta ,2}({\bar{\theta }}_{2}),\frac{\left(1+\alpha \right)}{4K\xi_{\alpha }(\xi_{\alpha }+4\gamma )}[(4K-l^{2})\xi_{\alpha }+(8K-3l^{2})\gamma -\sqrt{\Delta_{2}}]). \end{array}$$

where

$$\begin{array}{l} \xi_{\beta }=1-\beta -\beta \gamma ,\xi_{\alpha }=1+\alpha +\alpha \gamma \\ \Delta_{1}=64K^{2}\gamma^{2}+l^{4}{\left(\xi_{\beta }+3\gamma \right)}^{2}-8K\gamma l^{2}(\xi_{\beta }+6\gamma )>0\\ \Delta_{2}=64K^{2}\gamma^{2}+l^{4}{\left(\xi_{\alpha }+3\gamma \right)}^{2}-8K\gamma l^{2}(\xi_{\alpha }+6\gamma )>0 \end{array}$$

Given the complexity of the expression ${\bar{w}}_{\theta ,1}$, the equivalence relation ${\tilde{w}}_{\theta ,1}({\bar{\theta }}_{1})$ is expressed as shown above.

Another set of feasible boundary solutions for $\Pi_{\theta ,i}^{m}$ is to make *θ* = 0. Obviously, the CS-E contract would be the same as the wholesale price contract in this case; therefore, the optimal decision *θ* = 0 is reduced to an optimal solution under the wholesale price contract.

In addition, it is easy to demonstrate that the equilibrium solution on the boundary $\theta_{i}^{*}={\hat{\theta }}_{i}$ or *w* = *c* is not a feasible solution for the manufacturer.

The manufacturer's global optimal strategy can be resolved, and we provide the conclusion in Theorem 1.

**Theorem 1.** When only retailers have fairness concerns in the supply chain, the optimal strategy of manufacturers (w*,θ*) is shown in Table 2.

**Proof.** See Appendix B. □

**Table 2:** The manufacturer's optimal strategy with a cost-sharing contract.

Feasible region			(w*θ*)
$K>\frac{3}{4}l^{2}$	**$\beta \leq \frac{1-2\gamma }{1+\gamma }-\frac{3\gamma l^{2}}{2(4K-3l^{2})(1+\gamma )}\;and\;\alpha \geq \beta$**		**$\left(w_{\theta ,1}^{*},\theta_{1}^{*}\right)$**
	**$\frac{1-2\gamma }{1+\gamma }-\frac{3\gamma l^{2}}{2(4K-3l^{2})(1+\gamma )}<\beta <\frac{1}{1+\gamma }\;and\;\alpha \geq \max\left\{{\bar{\alpha }}_{s,1}(\beta ),\beta \right\}$**		**$\left({\bar{w}}_{\theta ,1}^{},{\bar{\theta }}_{1}^{}\right)$**
	**$\frac{1-2\gamma }{1+\gamma }-\frac{3\gamma l^{2}}{2(4K-3l^{2})(1+\gamma )}<\beta <\frac{1}{1+\gamma }\;and\;\beta \leq \alpha <{\bar{\alpha }}_{s,1}(\beta )$**		**$\left(w_{\theta ,3}^{*},\theta_{3}^{*}\right)$**
	**$\beta \geq \frac{1}{1+\gamma }\;and\;\alpha \geq Max\left\{\frac{\gamma -1}{1+\gamma }+\frac{\gamma l^{2}}{2(4K-2.5l^{2})(1+\gamma )},\beta \right\}$**		**$\left(w_{\theta ,2}^{*},\forall [0,{\hat{\theta }}_{2})\right)$**
	**$\beta \geq \frac{1}{1+\gamma }\;and\;\beta \leq \alpha <\frac{\gamma -1}{1+\gamma }+\frac{\gamma l^{2}}{2(4K-2.5l^{2})(1+\gamma )}$**		**$\left(w_{\theta ,3}^{*},\theta_{3}^{*}\right)$**
$\frac{1}{2}l^{2}<K\leq \frac{3}{4}l^{2}$	**$0<\beta <\frac{1}{1+\gamma },\beta \leq \alpha <{\bar{\alpha }}_{s,2}(\beta )\;and\;\gamma \geq \bar{\gamma }$**		**$\left(w_{3}^{*},0\right)$**
	**$0<\beta <\frac{1}{1+\gamma }$**	**$\alpha \geq \max\left\{{\bar{\alpha }}_{s,2}(\beta ),\beta \right\},\gamma \geq \bar{\gamma }$**	$\left({\bar{w}}_{\theta ,1}^{},{\bar{\theta }}_{1}^{}\right)$
		$\alpha \geq \beta ,\gamma <\bar{\gamma }$	
	**$\beta \geq \frac{1}{1+\gamma },\beta \leq \alpha <\frac{\gamma -1}{1+\gamma }\;and\;\gamma \geq \bar{\gamma }$**		**$\left(w_{3}^{*},0\right)$**
	**$\beta \geq \frac{1}{1+\gamma }$**	**$\alpha \geq \max\left\{\frac{\gamma -1}{1+\gamma },\beta \right\},\gamma \geq \bar{\gamma }$**	**$\left(w_{\theta ,2}^{*},\forall [0,{\hat{\theta }}_{2})\right)$**
		**$\alpha \geq \beta ,\gamma <\bar{\gamma }$**	

Note

${\bar{\alpha }}_{1}(\beta )=\arg\left\{\Pi_{\theta ,1}^{m}({\bar{w}}_{\theta ,1}^{*},{\bar{\theta }}_{1}^{*})=\Pi_{\theta ,3}^{m}(w_{\theta ,3}^{*},\theta_{3}^{*})\right\}=\frac{K{\left(a-c\right)}^{2}-(8K-4.5l^{2})\Pi_{\theta ,1}^{m}({\bar{w}}_{\theta ,1}^{*},{\bar{\theta }}_{1}^{*})}{(8K-4.5l^{2})(1+\gamma )\Pi_{\theta ,1}^{m}({\bar{w}}_{\theta ,1}^{*},{\bar{\theta }}_{1}^{*})-K{\left(a-c\right)}^{2}}$, ${\bar{\alpha }}_{2}(\beta )=\arg\left\{\Pi_{\theta ,1}^{m}({\bar{w}}_{\theta ,1}^{},{\bar{\theta }}_{1}^{})=\Pi_{\theta ,3}^{m}(w_{3}^{*},0)\right\}=\frac{K{\left(a-c\right)}^{2}-4(2K-l^{2})\Pi_{\theta ,1}^{m}({\bar{w}}_{\theta ,1}^{*},{\bar{\theta }}_{1}^{*})}{4(2K-l^{2})(1+\gamma )\Pi_{\theta ,1}^{m}({\bar{w}}_{\theta ,1}^{*},{\bar{\theta }}_{1}^{*})-K{\left(a-c\right)}^{2}}$

$\bar{\gamma }=\arg\left\{\Pi_{\theta ,3}^{m}({\bar{w}}_{\theta ,2},{\bar{\theta }}_{2})=\Pi_{\theta ,3}^{m}(w_{3}^{*},0)|\alpha =0\right\}$, $\bar{\gamma }\in [\frac{1}{2},+\infty )$.

As shown in Table 2, the manufacturers' decisions under a CS-E contract are not only affected by *α*,*β*,*γ* but also depend on the relations among the factors of the retailer's effort *K*,*l*. With $K=\frac{3}{4}l^{2}$ as the boundary, the manufacturers have two different sets of decisions to make. Manufacturers must therefore fully understand the external market environment of the retailers' sales efforts before designing the CS-E contract. In addition, a CS-E contract enables the members to achieve a "win-win" situation with maximum profit and utility for the channel when the manufacturer can guide the retailer to make decisions (*p*_*θ*,2_,*e*_*θ*,2_), which is the same as $\left(p_{c}^{*},e_{c}^{*}\right)$ for the strategy $\left(w_{\theta ,2}^{*},\forall [0,{\hat{\theta }}_{2})\right)$. By sorting Table 2, we obtain the following proposition.

**Proposition 5.** The CS-E contract enables a manufacturer to spontaneously achieve channel coordination with a fair-minded retailer if the relevant parameters are in the interval

$$\begin{array}{l} (I)\beta \geq \frac{1}{1+\gamma },\alpha \geq Max\left\{\frac{\gamma -1}{1+\gamma }+\frac{\gamma l^{2}}{2(4K-2.5l^{2})(1+\gamma )},\beta \right\}\;and\;K>\frac{3}{4}l^{2}\\ (II)\beta \geq \frac{1}{1+\gamma },\alpha \geq max\left\{\frac{\gamma -1}{1+\gamma },\beta \right\},\gamma \geq \bar{\gamma }\;and\;\frac{1}{2}l^{2}<K\leq \frac{3}{4}l^{2}\\ (III)\beta \geq \frac{1}{1+\gamma },\alpha \geq \beta ,\gamma <\bar{\gamma }\;and\;\frac{1}{2}l^{2}<K\leq \frac{3}{4}l^{2} \end{array}.$$

In the range $\frac{1}{2}l^{2}<K\leq \frac{3}{4}l^{2}$, the condition of channel coordination under a CS-E contract is more relaxed than the wholesale contract if retailers possess a lower benchmark of fairness $\gamma <\bar{\gamma }$, which means that the retailer thinks the manufacturer should be able to receive a higher share of the channel profits; otherwise, the two contracts would have the same ability to achieve channel coordination with a higher benchmark where $\gamma \geq \bar{\gamma }$. In the range $K>\frac{3}{4}l^{2}$, however, it is more difficult to coordinate the supply chain under a CS-E contract than under a wholesale price contract. Ultimately, the CS-E contract can provide maximum profit and maximum utility to the channel, but the conditions required for coordination are additional to the external environment of the retailer's market efforts and the fair benchmark, which differs from the wholesale price contracts.

However, there are also similarities between the two contracts in terms of coordinating the supply chain; that is, the retailer must have a strong sense of guilt and jealousy, which implies that the more the retailers pay attention to the fairness of the profit distribution, the more they can force the manufacturer to develop a strategy according to the expectations of the retailer. Meanwhile, this eliminates double marginalization in the channel, which enhances the consistency of the members' behaviour. Otherwise, the manufacturer will suffer from the retaliation of the retailer and hence reduce its own profit.

## Comparison of the wholesale price contract and the CS-E contract in a fair channel

In the previous section, we derived the global optimal strategy of the manufacturer under the two contracts and discussed the performance of the two contracts as it relates to the coordination of the supply chain. In this section, we focus on analysing the different effects of the two different mechanisms on three perspectives (the performance of the manufacturer, the retailer and the whole channel) under the same external parameters. Combining the characteristics of the decision-making interval under the two contracts, our analysis process is divided into four steps: (I) the comparison interval is divided into $K>\frac{3}{4}l^{2}$ and $\frac{1}{2}l^{2}<K\leq \frac{3}{4}l^{2}$; in addition, the effects of *γ* on the performance are further discussed under $\frac{1}{2}l^{2}<K\leq \frac{3}{4}l^{2}$; (II) the effect of the parameter *β* on supply chain performance under the two contracts is analysed and compared; (III) the effect of parameter *α* on supply chain performance under the two contracts is analysed and compared; and (IV) given that Tables 1 and 2 indicate that the optimal choice of the manufacturer is the local strategy with respect to the *α* interval or the *β* interval, the final comparison of the two contracts can be provided.

### The performance of the manufacturer

**Fig 1:**
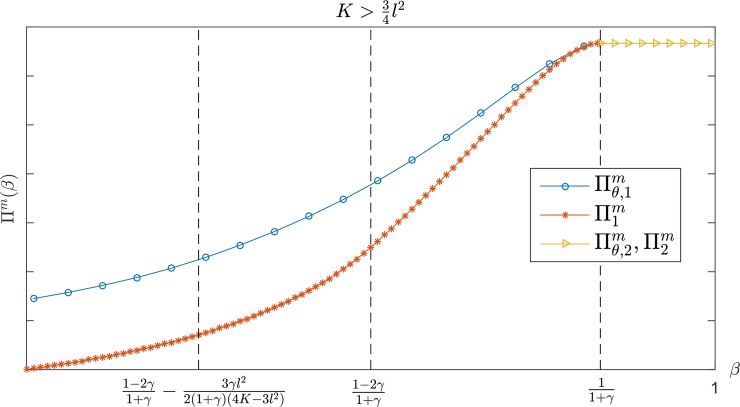
The manufacturer's profit with *β* in $K>\frac{3}{4}l^{2}$.

Fig 1 shows the local strategies of the manufacturer for parameter *β* when $K>\frac{3}{4}l^{2}$. In the range of $\beta <\frac{1}{1+\gamma }$, the manufacturer's profit is an increasing function of *β*, and the cost-sharing contract is better than the wholesale price contract. The gap in the manufacturer's profit is gradually narrowed with an increase of *β*. When the value of *β* exceeds $\frac{1}{1+\gamma }$, the manufacturer's profit remains a fixed value, and it is independent of the value of *β*. In this case, there is no difference between the two contracts. It is easy to demonstrate that in the interval of $\frac{1}{2}l^{2}<K\leq \frac{3}{4}l^{2}$, the local strategies of the manufacturer for *β* are also satisfied by the above conclusion; hence, the figure display in this case is omitted.

**Fig 2:**
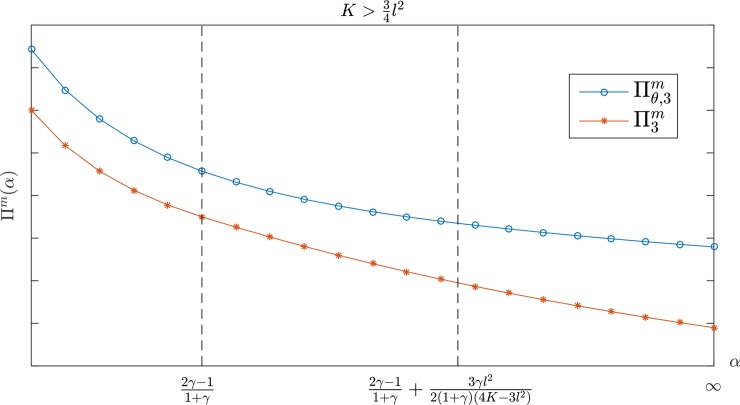
The manufacturer's profit with *α* in $K>\frac{3}{4}l^{2}$.

**Fig 3:**
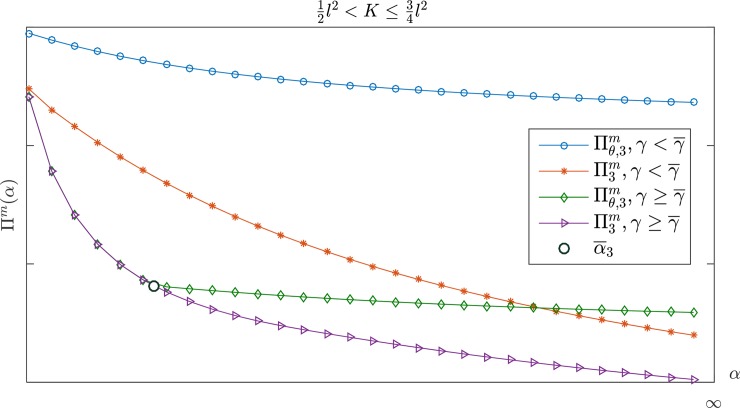
The manufacturer's profit with *α* in $\frac{1}{2}l^{2}<K\leq \frac{3}{4}l^{2}$.

Figs 2 and 3 show the changes in the manufacturer's profit when the retailer's jealousy concern is *α*. The manufacturer's profit is a monotone decreasing with *α*. Fig 2 shows that the manufacturer's profit under a CS-E contract is higher than the profit under a wholesale price contract if $K>\frac{3}{4}l^{2}$. When $\frac{1}{2}l^{2}<K\leq \frac{3}{4}l^{2}$, however, there are two possible scenarios, depending on the value of *γ* for the two contracts. As shown in Fig 3, the CS-E contract is always better than the wholesale price contract for the manufacturer's profit if $\gamma <\bar{\gamma }$. There is only one critical point ${\bar{\alpha }}_{3}$, ${\bar{\alpha }}_{3}<\frac{2\gamma -1}{1+\gamma }$. In the case of $\alpha <{\bar{\alpha }}_{3}$, the performance of the CS-E contract is the same as the wholesale price contract. The CS-E contract would be better than the wholesale price contract if $\alpha \geq {\bar{\alpha }}_{3}$. Here, ${\bar{\alpha }}_{3}$ is the only feasible solution for $\Pi_{\theta ,3}^{m}({\bar{w}}_{\theta ,3}^{},{\bar{\theta }}_{3}^{})=\Pi_{\theta ,3}^{m}(w_{3}^{*},0)$. For more details, please see Appendix B.

The conclusion above noted in Figs 1–3 indicates that when the retailer is guiltier, the manufacturer can obtain more profit; as the retailer becomes more jealous, the manufacturer can obtain more profit. Consequently, manufacturers are more willing to work with a retailer who has a larger *β* but smaller *α*. In other words, the manufacturer's monetary payoff consistently remains at a higher level regardless of whether the retailer chooses to be guilty or jealous. Conversely, if the retailer's *α* is small and *β* is large, the CS-E contract is more advantageous for the manufacturer even though his profit is at a lower level. Further, when the CS-E contract performance aligns with the wholesale price contract, the contract becomes unnecessary because the wholesale price contract is simpler. According to the manufacturer's optimal decision, which is illustrated in Tables 1 and 2, we can develop the following conclusions:

**Proposition 6.** When only the retailer cares about fairness in a dyadic supply chain, the CS-E contract can increase the profit of the manufacturer more than the wholesale price contract, except for the following intervals:

$$\begin{array}{l} (I)\beta \geq \frac{1}{1+\gamma },\alpha \geq Max\left\{\frac{\gamma -1}{1+\gamma }+\frac{\gamma l^{2}}{2(4K-2.5l^{2})(1+\gamma )},\beta \right\}\;and\;K>\frac{3}{4}l^{2}\\ (II)\beta \geq \frac{1}{1+\gamma },\alpha \geq \beta \;and\;\frac{1}{2}l^{2}<K\leq \frac{3}{4}l^{2}\\ (III)0<\beta <\frac{1}{1+\gamma },\beta \leq \alpha <{\bar{\alpha }}_{2}(\beta ),\gamma \geq \bar{\gamma }\;and\;\frac{1}{2}l^{2}<K\leq \frac{3}{4}l^{2} \end{array}.$$

## The performance of the retailer

### The profit and utility of the retailer

**Fig 4:**
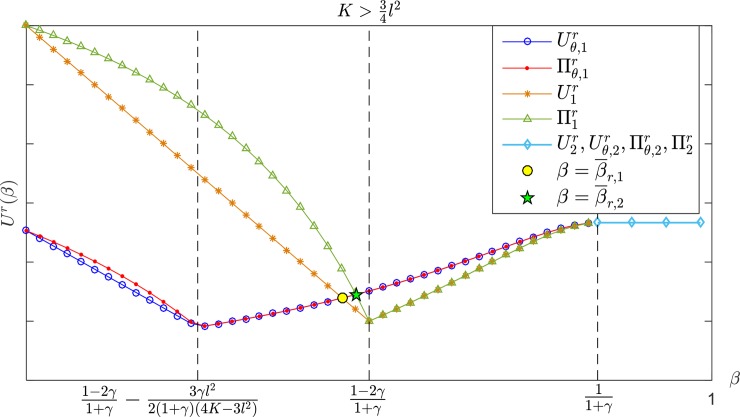
The retailer's profit and utility with *β* in $K>\frac{3}{4}l^{2}$.

As shown in Fig 4, when $K>\frac{3}{4}l^{2}$, the trend in the retailer's profit and utility first declines, then increases in the range $\beta <\frac{1}{1+\gamma }$ with *β*. The retailer's utility with a CS-E contract is higher (lower) than the wholesale price contract if ${\bar{\beta }}_{r,1}\leq \beta <\frac{1}{1+\gamma }$ ($0<\beta <{\bar{\beta }}_{r,1}$), while the retailer's profit under the CS-E contract is higher (lower) than the wholesale price contract if ${\bar{\beta }}_{r,2}\leq \beta <\frac{1}{1+\gamma }$ ($0<\beta <{\bar{\beta }}_{r,2}$), where ${\bar{\beta }}_{r,1}<{\bar{\beta }}_{r,2}<\frac{1-2\gamma }{1+\gamma }$ always holds. The same conclusion applies to the interval $\frac{1}{2}l^{2}<K\leq \frac{3}{4}l^{2}$.

**Fig 5:**
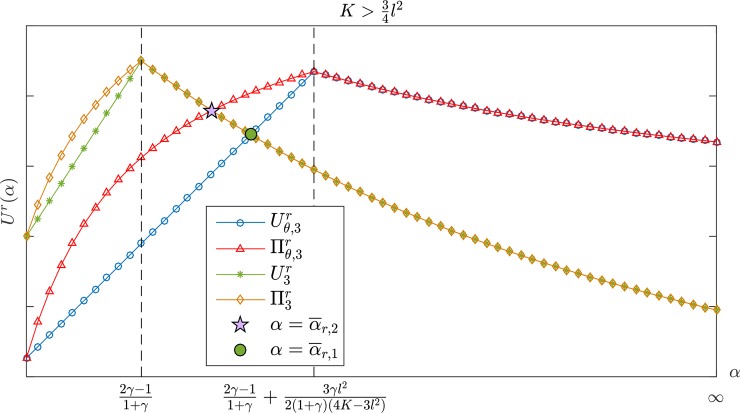
The retailer's profit and utility with *α* in $K>\frac{3}{4}l^{2}$.

**Fig 6:**
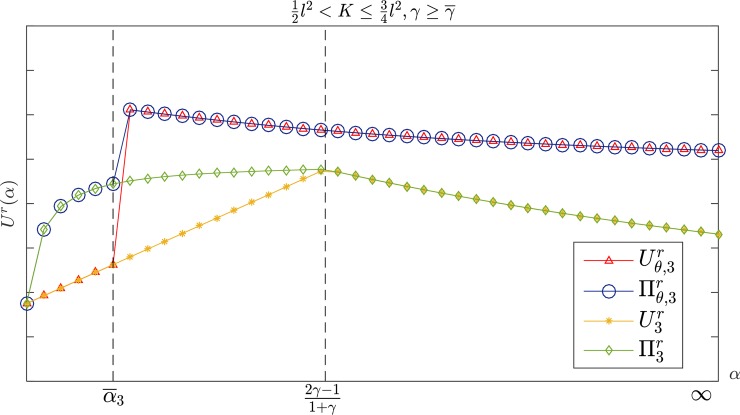
The retailer's profit and utility with *α* in $\frac{1}{2}l^{2}<K\leq \frac{3}{4}l^{2},\gamma \geq \bar{\gamma }$.

Fig 5 shows that when $K>\frac{3}{4}l^{2}$, the profit and utility of the retailer increases first and then decreases with *α*, and when $\alpha \geq {\bar{\alpha }}_{r,1}(\beta <\alpha <{\bar{\alpha }}_{r,1})$, it has $U_{\theta ,3}^{r}\geq U_{3}^{r}$ ($U_{\theta ,3}^{r}<U_{3}^{r}$), and $\Pi_{\theta ,3}^{r}\geq \Pi_{3}^{r}$ ($\Pi_{\theta ,3}^{r}<\Pi_{3}^{r}$) if $\alpha \geq {\bar{\alpha }}_{r,2}(\beta <\alpha <{\bar{\alpha }}_{r,2})$, where ${\bar{\alpha }}_{r,2}<{\bar{\alpha }}_{r,1}<\frac{2\gamma -1}{1+\gamma }+\frac{3\gamma l^{2}}{2(4K-3l^{2})(1+\gamma )}$ always holds. The retailer's profit and utility under the CS-E contract are both higher than those for the wholesale price contract if $\gamma <\bar{\gamma }$ or $\left\{\gamma \geq \bar{\gamma }\right\}\cap \left\{\alpha \geq {\bar{\alpha }}_{3}\right\}$ when $\frac{1}{2}l^{2}<K\leq \frac{3}{4}l^{2}$, which is shown in Fig 6. Otherwise, the two contracts have the same effect on the retailer. By sorting out the results above, we can draw the following conclusions:

**Proposition 7.** In a supply chain that is dominated by a selfish manufacturer, a fair retailer is able to obtain more utility with a CS-E contract than a wholesale price contract if

$$\begin{array}{l} (I){\bar{\beta }}_{r,1}\leq \beta <\frac{1}{1+\gamma },\alpha \geq Max\left\{Min\left\{{\bar{\alpha }}_{r,1},{\bar{\alpha }}_{s,1}(\beta )\right\},\beta \right\}\;and\;K>\frac{3}{4}l^{2}\\ (II){\bar{\beta }}_{r,1}\leq \beta <\frac{1}{1+\gamma },\alpha \geq Max\left\{{\bar{\alpha }}_{s,2}(\beta ),\beta \right\},\gamma \leq \bar{\gamma }\;and\;\frac{1}{2}l^{2}<K\leq \frac{3}{4}l^{2}\\ (III){\bar{\beta }}_{r,1}\leq \beta <\frac{1}{1+\gamma },\alpha \geq \beta ,\gamma >\bar{\gamma }\;and\;\frac{1}{2}l^{2}<K\leq \frac{3}{4}l^{2} \end{array}.$$

A fair retailer is able to obtain more profit with a CS-E contract than a wholesale contract if

$$\begin{array}{l} (I){\bar{\beta }}_{r,2}\leq \beta <\frac{1}{1+\gamma },\alpha \geq Max\left\{Min\left\{{\bar{\alpha }}_{r,2},{\bar{\alpha }}_{s,1}(\beta )\right\},\beta \right\}\;and\;K>\frac{3}{4}l^{2}\\ (II){\bar{\beta }}_{r,2}\leq \beta <\frac{1}{1+\gamma },\alpha \geq Max\left\{{\bar{\alpha }}_{s,2}(\beta ),\beta \right\},\gamma \leq \bar{\gamma }\;and\;\frac{1}{2}l^{2}<K\leq \frac{3}{4}l^{2}\\ (III){\bar{\beta }}_{r,2}\leq \beta <\frac{1}{1+\gamma },\alpha \geq \beta ,\gamma >\bar{\gamma }\;and\;\frac{1}{2}l^{2}<K\leq \frac{3}{4}l^{2} \end{array}.$$

In general, the utility or profit of the retailer is not reduced if the manufacturer's profit increases because of the CS-E contract. By contrast, the profit and the utility of the retailer can nevertheless be improved with an increase in the manufacturer's profit in some intervals, which can be inferred by combining the conclusions of Propositions 6 and 7.

## The sales effort of the retailer

Another important purpose of the CS-E contract for manufacturers concerned with the level of retailer effort is to improve the level of effort in the channel to increase market demand and potential channel competitiveness.

The level of retailers' efforts under the two contracts is similar to the variations in the manufacturer's profit. In accordance with Appendix A (a2) and Appendix B (b5), it can be demonstrated that the retailer's effort level under the cost-sharing contract is never lower than under the wholesale price contract, and the range of improvement is identical to that of the manufacturer's profit under the same conditions, which is shown in Proposition 6. It is not difficult to deduce that the CS-E contract is caused by an increase in the profits of the manufacturer, which is a good incentive mechanism for the retailer to provide more effort.

### The performance of the supply chain

**Fig 7:**
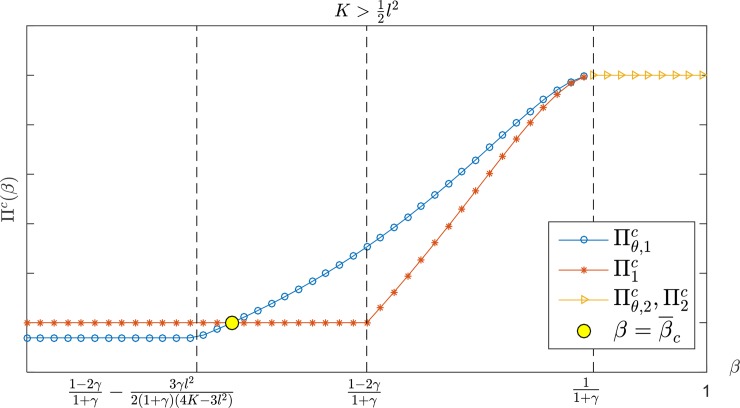
The channel's total profit with *β* in $K>\frac{3}{4}l^{2}$.

**Fig 8:**
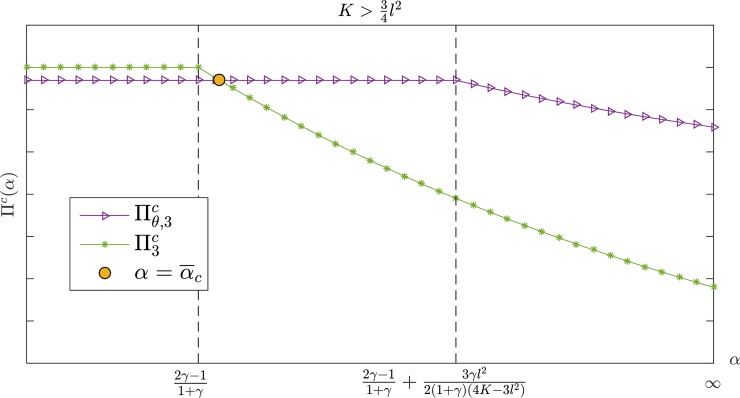
The channel's total profit with *α* in $K>\frac{3}{4}l^{2}$.

**Fig 9:**
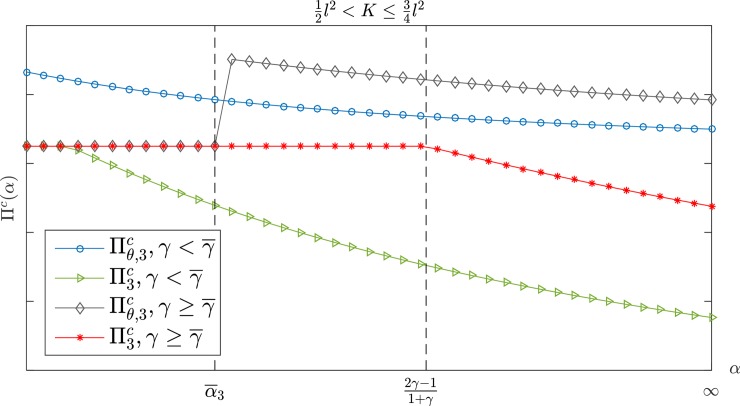
The channel's total profit with *α* in $\frac{1}{2}l^{2}<K\leq \frac{3}{4}l^{2}$.

Figs 7 and 8 show a comparison of the supply chain profit in $K>\frac{3}{4}l^{2}$. Moreover, $\Pi_{\theta ,i}^{c}\geq \Pi_{i}^{c}$ exists constantly if ${\bar{\beta }}_{c}\leq \beta <\frac{1}{1+\gamma },K>\frac{1}{2}l^{2}$ or $\alpha \geq {\bar{\alpha }}_{c},K>\frac{3}{4}l^{2}$ where $\Pi_{\theta ,i}^{c}$ and $\Pi_{i}^{c}$ represent the overall supply chain profit under the CS-E contract and the wholesale price contract, respectively. Fig 9 shows that $\Pi_{\theta ,i}^{c}$ is always higher than $\Pi_{i}^{c}$ when $\gamma <\bar{\gamma }$ or $\left\{\gamma \geq \bar{\gamma }\right\}\cap \left\{\alpha \geq {\bar{\alpha }}_{3}\right\}$ under the interval of $\frac{1}{2}l^{2}<K\leq \frac{3}{4}l^{2}$; otherwise, the two contracts have the same effect on the supply chain profit.

**Proposition 8.** In a supply chain that is dominated by manufacturers and only retailers have fairness concerns, the total profit of the supply chain under a CS-E contract is more than under a wholesale price contract if the supply chain meets the following conditions:

$$\begin{array}{l} (I){\bar{\beta }}_{c}\leq \beta <\frac{1}{1+\gamma },\alpha \geq Max\left\{Min\left\{{\bar{\alpha }}_{c},{\bar{\alpha }}_{s,1}\right\},\beta \right\}\;and\;K>\frac{3}{4}l^{2}\\ (II){\bar{\beta }}_{c}\leq \beta <\frac{1}{1+\gamma },\alpha \geq Max\left\{{\bar{\alpha }}_{s,2},\beta \right\},\gamma \leq \bar{\gamma }\;and\;\frac{1}{2}l^{2}<K\leq \frac{3}{4}l^{2}\\ (III){\bar{\beta }}_{c}\leq \beta <\frac{1}{1+\gamma },\alpha \geq \beta ,\gamma >\bar{\gamma }\;and\;\frac{1}{2}l^{2}<K\leq \frac{3}{4}l^{2} \end{array}.$$

By summarizing the relevant conclusions of Propositions 6–8, we can extend these conclusions to Proposition 9.

**Proposition 9.** In a supply chain dominated by manufacturers and only retailers have a fairness concern, the manufacturers' profits; the retailers' efforts, profits, and utilities; and the overall supply chain profit will be higher with a CS-E contract than with a wholesale price contract if the parameters of the supply chain meet the following conditions:

$$\begin{array}{l} (I){\bar{\beta }}_{r,2}\leq \beta <\frac{1}{1+\gamma },\alpha \geq Max\left\{Min\left\{{\bar{\alpha }}_{r,1},{\bar{\alpha }}_{s,1}\right\},\beta \right\}\;and\;K>\frac{3}{4}l^{2}\\ (II){\bar{\beta }}_{r,2}\leq \beta <\frac{1}{1+\gamma },\alpha \geq Max\left\{{\bar{\alpha }}_{s,2},\beta \right\},\gamma \leq \bar{\gamma }\;and\;\frac{1}{2}l^{2}<K\leq \frac{3}{4}l^{2}\\ (III){\bar{\beta }}_{r,2}\leq \beta <\frac{1}{1+\gamma },\alpha \geq \beta ,\gamma >\bar{\gamma }\;and\;\frac{1}{2}l^{2}<K\leq \frac{3}{4}l^{2} \end{array}$$

Proposition 9 indicates that a CS-E contract can be a better choice than a wholesale price contract if there is no fairness concern, but it may require retailers to have a high level of jealousy and moderate levels of guilt. For ‘indifferent’ retailers with low levels of *β* and *α*, selfish manufacturers will have no qualms about expanding their proportion of profits in a supply chain with an opportunity for sharing the cost of effort, and this will, of course, also lead to lower utility, lower profits for retailers, and even a decline in supply chain efficiency. The two contracts may be the same if retailers' fairness concerns and the environmental factors for sales effort meet the intervals shown in Proposition 6.

## Conclusions

The majority of previous research on channel strategies considering fairness have the following two characteristics: (a) a complete and complex model is introduced to describe players’ fairness, but the discussion is limited to a simple contract or single-variable dependent demand; and (b) various factors or a complex channel structure has been considered; however, the expression of fairness utility is simplified to facilitate the solution process. We addressed the issue of channel strategies between a manufacturer and a fair-minded retailer in which the market demand is influenced linearly by the sales efforts and the retail price. By deriving the equilibrium, we first examined the effects of fairness concerns on the channel performance based on our model with a simple wholesale price and a cost-sharing contract. Second, according to those equilibrium strategies, we analysed the influence of the retailer's fairness concern coefficient *α*,*β* and sales effort coefficient *K*,*l* on the performance of a fair supply chain in a separate interval for each of the two contracts and determined which contract was beneficial for which party and under which conditions.

This study reveals that (1) under the wholesale price contract, the retailer's sales effort will enhance the overall profit of the supply chain, although it has no direct influence on the manufacturer's choice of optimal decision; (2) under the cost-sharing contracts, the channel optimal decisions are not only affected by the factors that characterize the fairness concern of retailers but also involve the external environment of sales efforts; (3) both wholesale price and CS-E contracts can attain channel coordination in certain conditions, and the CS-E contract is more likely to make the channel coordinate when effort cost coefficient *K* is not large, and vice versa; (4) the CS-E contract consistently enhances the manufacturer's profit and the retailer's effort level more than the wholesale price contract when the supply chain is not able to coordinate; and (5) the utility and profit of a fair retailer and the profit of the entire supply chain are better under a CS-E contract than under a wholesale price contract if the retailer's sense of fairness is at a middle level, i.e., neither strong nor weak.

Based on the above results, some managerial implications may be derived. For example, our results help the manufacturing managers who are in leadership roles make optimal decisions and provide them with a better understanding of the impact of the retailer's fairness concerns. Further, by observing the characteristics of the retailer and the external environment, the manufacturer manager can clearly judge whether the cost-sharing contract must be used and how to use the cost-sharing contract to incentivize the retailer’s effort to enhance the channel profits or even what type of retailer should be selected. Conversely, the retailer's sense of fairness can significantly affect the profit of the channel, which in turn motivates the manufacturer to make decisions to maximize the profit of the channel. In addition, the CS-E contract has a certain enhancement effect on the retailer's performance, except for the retailer with a low consciousness of fairness that indicates that a simple wholesale price is a better choice.

From the policy implementation perspective, this study is limited in that the discussion of the cost-sharing contract relies on the assumption that the retailer’s effort decision is verifiable. However, a portion of the effort may be difficult to quantify in reality. The study of revenue-sharing contracts or revenue-sharing combined with cost-sharing contracts, therefore, may be more realistic in the context of future considerations. In addition, this study is based on a unilateral fairness concern and a unilateral effort by the retailer of the supply chain. Indeed, the manufacturer may also have a fairness consideration and quality effort. Finally, other areas worth studying involve uncertainty about market demand, asymmetric information, and supply chains with different structures, which could lead to different game sequences.

## Appendix A

### A.1 Manufacturer's decision regarding a wholesale price contract

To solve the sub-problem of Eq (13), the manufacturer's optimization problem under (*p*_1_(*w*),*e*_1_(*w*)) is

$$\begin{array}{l} \underset{p,e}{\max}(w-c)D(p_{1}(w),e_{1}(w))-\frac{K}{2}\theta e_{1}{\left(w\right)}^{2}\\ s.t.\;w\leq {\tilde{w}}_{1} \end{array}.$$ (a.1.1)

The optimal solution for the case (a.1.1) can be written as

$$w^{*}=\left\{ \begin{array}{l} w_{1}^{*}\;\mathrm{if}\;\beta <\frac{1-2\gamma }{1+\gamma }\\ {\tilde{w}}_{1}\;\mathrm{otherwise} \end{array}\right.$$

Similarly, the manufacturer's optimal solutions if the retailer chooses (*p*_2_(*w*),*e*_2_(*w*)) and (*p*_3_(*w*),*e*_3_(*w*)) are $w^{*}=\left\{ \begin{array}{l} {\tilde{w}}_{1}\;\mathrm{if}\;\beta <\frac{1}{1+\gamma }\\ w_{2}^{*}\;\mathrm{otherwise} \end{array}\right.$ and $w^{*}=\left\{ \begin{array}{l} w_{3}^{*}\;\mathrm{if}\;\alpha \leq \frac{2\gamma -1}{1+\gamma }\\ {\tilde{w}}_{2}\;\mathrm{otherwise} \end{array}\right.$, respectively.

We compared the manufacturer's profit in the intersection interval of *α*,*β* according to *α*≥*β*; then, we have the manufacturer's global optimal strategy and the feasible region, which are shown in Table 1.

### A.2 The retailer's performance for a wholesale price contract

The retailer's profit and utility that correspond to the manufacturer's optimal strategy w* is presented below:

$$\left\{ \begin{array}{l} U_{1}^{r}=\frac{K(1-\beta ){\left(a-c\right)}^{2}}{8(2K-l^{2})}\;\Pi_{1}^{r}=\frac{K{\left(a-c\right)}^{2}(1-\beta -\beta \alpha \gamma )}{8(2K-l^{2})(1-\beta -\beta \gamma )}\\ \;if\;w^{*}=w_{1}^{*}\\ U_{1}^{r}=\Pi_{1}^{r}=\frac{2K\gamma^{2}(1-\beta ){\left(a-c\right)}^{2}}{(2K-l^{2}){\left(1-\beta -\beta \gamma +2\gamma \right)}^{2}}\\ \;if\;w^{*}={\tilde{w}}_{1}\\ U_{2}^{r}=\Pi_{2}^{r}=\frac{K\gamma {\left(a-c\right)}^{2}}{2(1+\gamma )(2K-l^{2})}\\ \;if\;w^{*}=w_{2}^{*}\\ U_{3}^{r}=\frac{K(1+\alpha ){\left(a-c\right)}^{2}}{8(2K-l^{2})}\;\Pi_{3}^{r}=\frac{K{\left(a-c\right)}^{2}(1+\alpha +3\alpha \gamma )}{8(2K-l^{2})(1+\alpha +\alpha \gamma )}\\ \;if\;w^{*}=w_{3}^{*}\\ U_{3}^{r}=\Pi_{3}^{r}=\frac{2K\gamma^{2}(1+\alpha ){\left(a-c\right)}^{2}}{(2K-l^{2}){\left(1+\alpha +\alpha \gamma +2\gamma \right)}^{2}}\\ \;if\;w^{*}={\tilde{w}}_{2} \end{array}\right.$$ (a.2.1)

The retailer's sales efforts that correspond to the manufacturer's optimal strategy w* are presented below:

$$\left\{ \begin{array}{l} e_{1}^{r}=\frac{l(a-c)}{2(2K-l^{2})}\;if\;w^{*}=w_{1}^{*}\\ e_{1}^{r}=\frac{2\gamma l(a-c)}{\left(2K-l^{2})(1-\beta -\beta \gamma +2\gamma \right)}\;if\;w^{*}={\tilde{w}}_{1}\\ e_{2}^{r}=\frac{l(a-c)}{2K-l^{2}}\;if\;w^{*}=w_{2}^{*}\\ e_{3}^{r}=\frac{l(a-c)}{2(2K-l^{2})}\;if\;w^{*}=w_{3}^{*}\\ e_{3}^{r}=\frac{2\gamma l(a-c)}{\left(2K-l^{2})(1+\alpha +\alpha \gamma +2\gamma \right)}\;if\;w^{*}={\tilde{w}}_{2} \end{array}\right.$$ (a.2.2)

## Appendix B

### B.1 Manufacturer's decision regarding a CS-E contract

This is similar to the solution for A1. The manufacturer's decision-making process can be divided into two steps: first, the local maximum profit that the manufacturer may obtain corresponding to the local decision of the retailer is solved, and second, the manufacturer's local equilibrium strategies are compared to determine his global optimal strategy.

### B.1.1 The local optimal strategy of the manufacturer under (*p*_*θ*,1_,*e*_*θ*,1_)

The local optimal problem of the manufacturer is

$$\begin{array}{l} \underset{w,\theta }{\max}(w-c)D(p_{\theta ,1},e_{\theta ,1})-\frac{K}{2}\theta {\left(e_{\theta ,1}\right)}^{2}\\ s.t.\;\left\{ \begin{array}{l} w(\theta )\leq {\tilde{w}}_{1}(\theta )\\ \theta <\min\left\{{\hat{\theta }}_{1},1\right\}\;\mathrm{or}\;{\hat{\theta }}_{1}\leq \theta <1 \end{array}\right. \end{array}.$$ (b.1.1)

The equilibrium strategy of the manufacturer and the corresponding profit without constraints in (b.1.1) are $w_{\theta ,1}^{*}=\frac{\left(1-\beta )[(8K-3l^{2})a+2(4K-3l^{2})c\right]}{\left(1-\beta -\beta \gamma )(16K-9l^{2}\right)}-\frac{\beta \gamma c}{\left(1-\beta -\beta \gamma \right)}$, $\theta_{1}^{*}=\frac{\left(1-\beta \right)}{3(1-\beta -\beta \gamma )}$, and $\Pi_{\theta ,1}^{m}=\frac{2K(1-\beta ){\left(a-c\right)}^{2}}{\left(1-\beta -\beta \gamma )(16K-9l^{2}\right)}$.

According to the constraints, we can obtain the following results:

$$w_{\theta ,1}^{*}\leq {\tilde{w}}_{1}(\theta_{1}^{*})\Rightarrow \beta \leq \frac{1-2\gamma }{1+\gamma }-\frac{3\gamma l^{2}}{2(1+\gamma )(4K-3l^{2})},\theta_{1}^{*}<{\hat{\theta }}_{1}\Rightarrow 4K>3l^{2},\theta_{1}^{*}<1\Rightarrow \beta <\frac{2}{2+3\gamma }.$$

Because $\frac{2}{2+3\gamma }>\frac{1-2\gamma }{1+\gamma }-\frac{3\gamma l^{2}}{2(1+\gamma )(4K-3l^{2})}$, the $\left(w_{\theta ,1}^{*},\theta_{1}^{*}\right)$ strategy can be chosen if it satisfies the constraint condition $\left\{K>\frac{3}{4}l^{2}\right\}\cap \left\{\beta \leq \frac{1-2\gamma }{1+\gamma }-\frac{3\gamma l^{2}}{2(1+\gamma )(4K-3l^{2})}\right\}$.

The manufacturer's optimal strategy is $w={\tilde{w}}_{1}(\theta )$ or *θ* = 0 if $\left(w_{\theta ,1}^{*},\theta_{1}^{*}\right)$ cannot satisfy any constraint in (b.1.1).

When $w={\tilde{w}}_{1}(\theta )$, (b.1.1) can be written as

$$\begin{array}{l} \Pi_{\theta ,1}^{m}=\underset{\theta }{Max}({\tilde{w}}_{1}(\theta )-c)D(p_{\theta ,1}({\tilde{w}}_{1}(\theta ),\theta ),e_{\theta ,1}({\tilde{w}}_{1}(\theta ),\theta ))-\frac{K}{2}\theta {\left(e_{\theta ,1}({\tilde{w}}_{1}(\theta ),\theta )\right)}^{2}\\ s.t.\;\theta <\min\left\{{\hat{\theta }}_{1},1\right\}\;\mathrm{or}\;{\hat{\theta }}_{1}\leq \theta <1 \end{array}$$ (b.1.2)

Thus, we obtain the following two possible *θ*:

$$\theta_{1}^{-,+}=\frac{\left(1-\beta \right)}{4K(1-\beta -\beta \gamma +4\gamma )(1-\beta -\beta \gamma )}[(4K-l^{2})(1-\beta -\beta \gamma )+(8K-3l^{2})\gamma \mp \sqrt{\Delta_{1}}],$$

where Δ_1_ has already been explained in the previous sections and $\theta_{1}^{+}$ should be abandoned since it is the minimum point for (b.1.2).

Then, there are several results:

$\theta_{1}^{-}>0\Rightarrow \left\{ \begin{array}{l} 2K>l^{2}\\ \beta <\frac{1}{1+\gamma } \end{array}\right.$; therefore, $\theta_{1}^{-}\in [0,{\hat{\theta }}_{1})$. Additionally, it is easy to prove that $\theta_{1}^{-}<{\hat{\theta }}_{1}$ always holds.

Thus, ${\bar{\theta }}_{1}=\theta_{1}^{-}=\frac{\left(1-\beta \right)}{4K(1-\beta -\beta \gamma +4\gamma )(1-\beta -\beta \gamma )}[(4K-l^{2})(1-\beta -\beta \gamma )+(8K-3l^{2})\gamma -\sqrt{\Delta_{1}}]$.

The manufacturer's profit with ${\bar{\theta }}_{1}$ can be given as

$$\Pi_{\theta ,1}^{m}({\bar{w}}_{\theta ,1},{\bar{\theta }}_{1})=2(1-\beta ){\left(a-c\right)}^{2}\cdot \frac{F_{1}(\tau )+L_{1}(\tau )\sqrt{\Delta_{1}}}{{\left(F_{2}(\tau )+L_{2}(\tau )\sqrt{\Delta_{1}}\right)}^{2}}.$$

where *τ* = *β*

$$\begin{array}{l} F_{1}(\tau )=\frac{{\left(1-\tau -\tau \gamma +3\gamma \right)}^{3}l^{8}}{4096}+\frac{(1-\tau -\tau \gamma +3\gamma ){\left(1-\tau -\tau \gamma \right)}^{2}Kl^{6}}{2048}-\frac{3\gamma {\left(1-\tau -\tau \gamma \right)}^{2}K^{2}l^{6}}{512}-K^{3}\gamma^{3}(K-\frac{l^{2}}{2})\\ F_{2}(\tau )=\frac{1}{32}l^{4}{\left(1-\tau -\tau \gamma +3\gamma \right)}^{2}-\gamma (K-\frac{1}{4}l^{2})(1-\tau -\tau \gamma +2\gamma )\\ L_{1}(\tau )=\frac{{\left(1-\tau -\tau \gamma +3\gamma \right)}^{2}l^{6}}{4096}-\frac{{\left(1+\gamma \right)}^{2}\tau^{2}+2(\gamma +1)(\gamma -1)-12\gamma^{2}-2\gamma +1}{2048}-\frac{\gamma^{2}K^{2}(16K+(1-\tau -\tau \gamma -2\gamma )l^{2})}{128}\\ L_{2}(\tau )=\frac{1}{32}l^{2}(1-\tau -\tau \gamma +3\gamma )-\frac{1}{8}K(1-\tau -\tau \gamma +2\gamma ). \end{array}$$

According to the above analysis, the equilibrium strategy of the manufacturer on the boundary $w={\tilde{w}}_{1}(\theta )$ can be reduced to

$$\begin{array}{l} \Pi_{\theta ,1}^{m}({\bar{w}}_{\theta ,1},{\bar{\theta }}_{1})=2(1-\beta ){\left(a-c\right)}^{2}\cdot \frac{F_{1}(\tau )+L_{1}(\tau )\sqrt{\Delta_{1}}}{{\left(F_{2}(\tau )+L_{2}(\tau )\sqrt{\Delta_{1}}\right)}^{2}}\\ s.t.\;\left\{\left\{\beta >\frac{1-2\gamma }{1+\gamma }-\frac{3\gamma l^{2}}{2(1+\gamma )(4K-3l^{2})}\right\}\cap \left\{4K>3l^{2}\right\}\right\}\cup \left\{\frac{1}{2}l^{2}<K\leq \frac{3}{4}l^{2}\right\} \end{array}.$$

When *θ*= 0, it is easy to find the optimal wholesale price *w* in this scenario, which is $w=w_{1}^{*}=\frac{(1-\beta )(a+c)-2\beta \gamma c}{2(1-\beta -\beta \gamma )}$, where $w_{1}^{*}$ can be found in the previous problem of the wholesale price contract, and the profit with $w_{1}^{*}$ is $\Pi_{\theta ,1}^{m}(w_{1}^{*},0)=\frac{K(1-\beta ){\left(a-c\right)}^{2}}{4(1-\beta -\beta \gamma )(2K-l^{2})}$. Then, $w_{1}^{*}\leq {\tilde{w}}_{\theta ,1}(\theta =0)\Rightarrow \beta \leq \frac{1-2\gamma }{1+\gamma }$; thus, the equilibrium strategy of the manufacturer on the boundary $\left(w_{1}^{*},0\right)$ can be reduced to

$$\begin{array}{l} \Pi_{\theta ,1}^{m}(w_{1}^{*})=\frac{K(1-\beta ){\left(a-c\right)}^{2}}{4(1-\beta -\beta \gamma )(2K-l^{2})}\\ s.t.\;\left\{\left\{\frac{1-2\gamma }{1+\gamma }-\frac{3\gamma l^{2}}{2(1+\gamma )(4K-3l^{2})}<\beta \leq \frac{1-2\gamma }{1+\gamma }\right\}\cap \left\{K>\frac{3}{4}l^{2}\right\}\right\}\cup \{\left\{\frac{1}{2}l^{2}<K\leq \frac{3}{4}l^{2}\right\}\cap \left\{0<\beta \leq \frac{1-2\gamma }{1+\gamma }\right\}\}. \end{array}$$

Obviously, the manufacturer's profit $\Pi_{\theta ,1}^{m}(w_{1}^{*},0)$ and $\Pi_{\theta ,1}^{m}({\bar{w}}_{\theta ,1},{\bar{\theta }}_{1})$ should be compared to the overlapping interval $\left\{\frac{1}{2}l^{2}<K\leq \frac{3}{4}l^{2}\right\}\cap \left\{0<\beta \leq \frac{1-2\gamma }{1+\gamma }\right\}$. Note that $0<\beta \leq \frac{1-2\gamma }{1+\gamma }\Rightarrow 0<\gamma <\frac{1}{2}$. In this interval, $\frac{\partial \Pi_{\theta ,1}^{m}({\bar{w}}_{\theta ,1},{\bar{\theta }}_{1})}{\partial \beta }>0$ and $\frac{\partial \Pi_{\theta ,1}^{m}(w_{1}^{*},0)}{\partial \beta }>0$ always hold.

Let $T(\beta )=\Pi_{\theta ,1}^{m}({\bar{w}}_{\theta ,1},{\bar{\theta }}_{1})-\Pi_{\theta ,1}^{m}(w_{1}^{*},0)$; then we can obtain

1. *T*(*β*)>0 if $\beta =\frac{1-2\gamma }{1+\gamma }$ and
2. $\frac{\partial T(\beta )}{\partial \gamma }<0$ if *β* = 0. Moreover, $\left\{ \begin{array}{l} T(\beta =0,\gamma =0)>0\\ T(\beta =0,\gamma =\frac{1}{2})>0 \end{array}\right.\Rightarrow T(\beta =0,0<\gamma <\frac{1}{2})>0$.

Thus, $\Pi_{\theta ,1}^{m}({\bar{w}}_{\theta ,1},{\bar{\theta }}_{1})-\Pi_{\theta ,1}^{m}(w_{1}^{*},0)>0$, which indicates that the manufacturer will never choose the strategy $\left(w_{1}^{*},0\right)$.

Based on the above analysis, the local optimal strategy of the manufacturer for the retailer's decision (*p*_θ,1_,*e*_*θ*,1_) can be expressed as

$$\left\{ \begin{array}{l} (w,\theta )=(w_{\theta ,1}^{*},\theta_{1}^{*})\Pi_{\theta ,1}^{m}=\frac{2K(1-\beta ){\left(a-c\right)}^{2}}{\left(1-\beta -\beta \gamma )(16K-9l^{2}\right)}\\ \;\mathrm{if}\;\left\{K>\frac{3}{4}l^{2}\right\}\cap \left\{\beta \leq \frac{1-2\gamma }{1+\gamma }-\frac{3\gamma l^{2}}{2(1+\gamma )(4K-3l^{2})}\right\}\\ (w,\theta )=({\bar{w}}_{\theta ,1},{\bar{\theta }}_{1})\Pi_{\theta ,1}^{m}=2(1-\beta ){\left(a-c\right)}^{2}\cdot \frac{F_{1}(\tau )+L_{1}(\tau )\sqrt{\Delta_{1}}}{{\left(F_{2}(\tau )+L_{2}(\tau )\sqrt{\Delta_{1}}\right)}^{2}}\\ \;\mathrm{if}\;\left\{\left\{\beta >\frac{1-2\gamma }{1+\gamma }-\frac{3\gamma l^{2}}{2(1+\gamma )(4K-3l^{2})}\right\}\cap \left\{4K>3l^{2}\right\}\right\}\cup \left\{\frac{1}{2}l^{2}<K\leq \frac{3}{4}l^{2}\right\} \end{array}\right..$$ (b.1.3)

### B.1.2 The local optimal strategy of the manufacturer under (*p*_θ,2_,*e*_*θ*,2_)

The local optimal problem of the manufacturer is

$$\begin{array}{l} \underset{w,\theta }{\max}(w-c)D(p_{\theta ,2},e_{\theta ,2})-\frac{K}{2}\theta {\left(e_{\theta ,2}\right)}^{2}\\ s.t.\;\left\{ \begin{array}{l} {\tilde{w}}_{1}(\theta )<w(\theta )\leq {\tilde{w}}_{2}(\theta )\\ \theta <{\hat{\theta }}_{2}\\ \Pi_{}^{r}=\gamma \Pi_{}^{s} \end{array}\right. \end{array}.$$ (b.1.4)

Based on the equation *Π*^*r*^(*p*_θ,2_,*e*_*θ*,2_) = *γΠ*^*m*^(*p*_θ,2_,*e*_*θ*,2_), it can be derived that for *w*,*θ*, $w(\theta )=\frac{\left[2K-l^{2}(1-\theta -\theta \gamma )](a-c\right)}{2(2K-l^{2})(1+\gamma )}+c$. Then, by substituting this relation into the manufacturer's profit problem (b.1.4), we can directly obtain the maximum profit in this scenario, which is $\Pi_{\theta ,2}^{m}=\frac{K{\left(a-c\right)}^{2}}{2(1+\gamma )(2K-l^{2})}$.

According to ${\tilde{w}}_{1}(\theta )<w_{\theta ,2}^{*}(\theta )\leq {\tilde{w}}_{2}(\theta )$, $\frac{\left[2K-l^{2}(1-\theta -\theta \gamma )](a-c\right)}{2(2K-l^{2})(1+\gamma )}+c\leq {\tilde{w}}_{2}(\theta )$ constantly holds for $\forall [0,{\hat{\theta }}_{2}]$, and we can obtain the condition $\beta \geq \frac{1}{1+\gamma }$ by the other side ${\tilde{w}}_{1}(\theta )\leq \frac{\left[2K-l^{2}(1-\theta -\theta \gamma )](a-c\right)}{2(2K-l^{2})(1+\gamma )}+c$. Note that ${\hat{\theta }}_{2}>0$ asks for 2*K*>*l*^2^.

In conclusion, the local optimal strategy of the manufacturer for the retailer's decision (*p*_θ,1_,*e*_*θ*,1_) may be expressed as

$$\left\{ \begin{array}{l} (w,\theta )=(w_{\theta ,2}^{*},\theta_{2}^{*})\Pi_{\theta ,2}^{m}=\frac{K{\left(a-c\right)}^{2}}{2(1+\gamma )(2K-l^{2})}\\ \;\mathrm{if}\;\left\{\beta \geq \frac{1}{1+\gamma }\right\}\cap \left\{K>\frac{1}{2}l^{2}\right\}\\ (w,\theta )=({\bar{w}}_{\theta ,1},{\bar{\theta }}_{1})\Pi_{\theta ,1}^{m}=2(1-\beta ){\left(a-c\right)}^{2}\cdot \frac{F_{1}(\tau )+L_{1}(\tau )\sqrt{\Delta_{1}}}{{\left(F_{2}(\tau )+L_{2}(\tau )\sqrt{\Delta_{1}}\right)}^{2}}\\ \;\mathrm{if}\;\left\{\beta <\frac{1}{1+\gamma }\right\}\cap \left\{K>\frac{1}{2}l^{2}\right\} \end{array}\right..$$ (b.1.5)

### B.1.3 The local optimal strategy of the manufacturer under (*p*_θ,3_,*e*_*θ*,3_)

The local optimal problem of the manufacturer is

$$\begin{array}{l} \underset{w,\theta }{\max}(w-c)D(p_{\theta ,3},e_{\theta ,3})-\frac{K}{2}\theta {\left(e_{\theta ,3}\right)}^{2}\\ s.t.\;\left\{ \begin{array}{l} w(\theta )\geq {\tilde{w}}_{2}(\theta )\\ 0\leq \theta <{\hat{\theta }}_{3} \end{array}\right. \end{array}.$$ (b.1.6)

Similar to the analysis method in B.1.1, we first derive the unconstrained optimal strategy $\left(w_{\theta ,3}^{*},\theta_{3}^{*}\right)$ of the manufacturer.

$w_{\theta ,3}^{*}=\frac{\left(1+\alpha )[(8K-3l^{2})a+2(4K-3l^{2})c\right]}{\left(1+\alpha +\alpha \gamma )(16K-9l^{2}\right)}+\frac{\alpha \gamma c}{\left(1+\alpha +\alpha \gamma \right)}$, and $\theta_{3}^{*}=\frac{\left(1+\alpha \right)}{3(1+\alpha +\alpha \gamma )}$; then, $\Pi_{\theta ,3}^{m}(w_{\theta ,3}^{*},\theta_{3}^{*})=\frac{2K(1+\alpha ){\left(a-c\right)}^{2}}{\left(1+\alpha +\alpha \gamma )(16K-9l^{2}\right)}$.

Accordingly, $w_{\theta ,3}^{*}\geq {\tilde{w}}_{2}(\theta_{3}^{*})\Rightarrow \alpha \leq \frac{2\gamma -1}{1+\gamma }+\frac{3\gamma l^{2}}{2(1+\gamma )(4K-3l^{2})}$, $\theta_{3}^{*}<{\hat{\theta }}_{3}\Rightarrow 4K>3l^{2}$; hence, the limitation of the manufacturer's strategy $\left(w_{\theta ,3}^{*},\theta_{3}^{*}\right)$ is $\left\{\alpha \leq \frac{2\gamma -1}{1+\gamma }+\frac{3\gamma l^{2}}{2(1+\gamma )(4K-3l^{2})}\right\}\cap \left\{4K>3l^{2}\right\}$.

Subsequently, we analysed the two possible boundary optimal strategies of the manufacturer.

When $w={\tilde{w}}_{2}(\theta )$, then ${\bar{\theta }}_{2}=\frac{\left(1+\alpha \right)}{4K(1+\alpha +\alpha \gamma +4\gamma )(1+\alpha +\alpha \gamma )}[(4K-l^{2})(1+\alpha +\alpha \gamma )+(8K-3l^{2})\gamma -\sqrt{\Delta_{2}}]<{\hat{\theta }}_{3}$, and $\Pi_{\theta ,3}^{s}({\bar{w}}_{\theta ,2},{\bar{\theta }}_{2})=2(1+\alpha ){\left(a-c\right)}^{2}\cdot \frac{F_{1}(\tau )+L_{1}(\tau )\sqrt{\Delta }}{{\left(F_{2}(\tau )+L_{2}(\tau )\sqrt{\Delta }\right)}^{2}}$ where *τ*= −*α*.

This conclusion is summarized as follows:

$$\begin{array}{l} \Pi_{\theta ,3}^{m}=\Pi_{\theta ,3}^{m}({\bar{w}}_{\theta ,2},{\bar{\theta }}_{2})=2(1+\alpha ){\left(a-c\right)}^{2}\cdot \frac{F_{1}(\tau )+L_{1}(\tau )\sqrt{\Delta }}{{\left(F_{2}(\tau )+L_{2}(\tau )\sqrt{\Delta }\right)}^{2}}\\ s.t.\left\{\left\{\alpha >\frac{2\gamma -1}{1+\gamma }+\frac{3\gamma l^{2}}{2(1+\gamma )(4K-3l^{2})}\right\}\cap \left\{4K>3l^{2}\right\}\right\}\cup \left\{\frac{1}{2}<K\leq \frac{3}{4}l^{2}\right\} \end{array}.$$

When *θ* = 0, the corresponding wholesale price is $w_{3}^{*}=\frac{(1+\alpha )(a+c)+2\alpha \gamma c}{2(1+\alpha +\alpha \gamma )}$, and the profit is $\Pi_{\theta ,3}^{m}(w_{3}^{*},0)=\frac{K(1+\alpha ){\left(a-c\right)}^{2}}{4(1+\alpha +\alpha \gamma )(2K-l^{2})}$.

Then, we have $\alpha \leq \frac{2\gamma -1}{1+\gamma }$ through $w_{3}^{*}\geq {\tilde{w}}_{2}(\theta =0)$, and note that $\frac{2\gamma -1}{1+\gamma }<\frac{2\gamma -1}{1+\gamma }+\frac{3\gamma l^{2}}{2(1+\gamma )(4K-3l^{2})}$ if 4*K*>3*l*^2^; thus, the manufacturer may consider the decision $\left(w_{3}^{*},0\right)$ only in a situation in which 4*K*≤3*l*^2^ for $\Pi_{\theta ,3}^{m}(w_{3}^{*},0)<\Pi_{\theta ,3}^{m}(w_{\theta ,3}^{*},\theta_{3}^{*})$. The above process may be summarized as follows:

$$\begin{array}{l} \Pi_{\theta ,3}^{m}=\Pi_{\theta ,3}^{m}(w_{3}^{*},0)=\frac{K(1+\alpha ){\left(a-c\right)}^{2}}{4(1+\alpha +\alpha \gamma )(2K-l^{2})}\\ s.t.\;\left\{0<\alpha \leq \frac{2\gamma -1}{1+\gamma }\right\}\cap \left\{4K\leq 3l^{2}\right\} \end{array}.$$

Then, we need to compare the manufacturer's profit $\Pi_{\theta ,3}^{m}(w_{3}^{*},0)$ and $\Pi_{\theta ,3}^{m}({\bar{w}}_{\theta ,2},{\bar{\theta }}_{2})$ to their overlapping region $\left\{0<\alpha \leq \frac{2\gamma -1}{1+\gamma }\right\}\cap \left\{4K\leq 3l^{2}\right\}$. Here, $0<\alpha \leq \frac{2\gamma -1}{1+\gamma }\Rightarrow \gamma >\frac{1}{2}$ should be noted.

In the region $\left\{0<\alpha \leq \frac{2\gamma -1}{1+\gamma }\right\}\cap \left\{4K\leq 3l^{2}\right\}$, it is not difficult to prove that $\frac{\partial \Pi_{\theta ,3}^{m}({\bar{w}}_{\theta ,2},{\bar{\theta }}_{2})}{\partial \alpha }<0$ and $\frac{\partial \Pi_{\theta ,3}^{m}(w_{3}^{*},0)}{\partial \alpha }<0$.

Let $R(\alpha )=\Pi_{\theta ,3}^{m}({\bar{w}}_{\theta ,2},{\bar{\theta }}_{2})-\Pi_{\theta ,3}^{m}(w_{3}^{*},0)$; when *α* = 0, then $\frac{\partial R(\alpha =0|\gamma >0)}{\partial \gamma }<0$ and $\left\{ \begin{array}{l} R(\alpha =0,\gamma =\frac{1}{2})>0\\ R(\alpha =0,\gamma \to +\infty )<0 \end{array}\right.$ because $R(\alpha =\frac{2\gamma -1}{1+\gamma })>0$ always holds; thus, we can determine that there is one and only one point $\bar{\gamma }$ that could make $\left\{ \begin{array}{l} R(\alpha =0)\geq 0\mathrm{if}\frac{1}{2}<\gamma \leq \bar{\gamma }\\ R(\alpha =0)<0\mathrm{if}\gamma >\bar{\gamma } \end{array}\right.$ in the range $\gamma \in (\frac{1}{2},+\infty )$.

Accordingly, $\Pi_{\theta ,3}^{m}({\bar{w}}_{\theta ,2},{\bar{\theta }}_{2})\geq \Pi_{\theta ,3}^{m}(w_{3}^{*},0)$ if $\frac{1}{2}<\gamma \leq \bar{\gamma }$, and when $\gamma >\bar{\gamma }$, there is one and only one point ${\bar{\alpha }}_{3}^{}$ that could render $\Pi_{\theta ,3}^{m}(w_{3}^{*},0)\geq \Pi_{\theta ,3}^{m}({\bar{w}}_{\theta ,2},{\bar{\theta }}_{2})$ if $0<\alpha \leq {\bar{\alpha }}_{3}^{}$ whereas $\Pi_{\theta ,3}^{m}(w_{3}^{*})<\Pi_{\theta ,3}^{m}({\bar{w}}_{\theta ,2},{\bar{\theta }}_{2})$ if $\alpha >{\bar{\alpha }}_{3}^{}$. Further, ${\bar{\alpha }}_{3}^{}<\frac{2\gamma -1}{1+\gamma }$ and ${\bar{\alpha }}_{3}^{}=0$ can be obtained if $\gamma =\bar{\gamma }$, which was discussed above.

In conclusion, the optimal decision of the manufacturer when confronted with retailer decision (*p*_θ,3_,*e*_*θ*,3_) can be expressed as

$$\left\{ \begin{array}{l} (w,\theta )=(w_{\theta ,3}^{*},\theta_{3}^{*})\Pi_{\theta ,3}^{m}=\frac{2K(1+\alpha ){\left(a-c\right)}^{2}}{\left(1+\alpha +\alpha \gamma )(16K-9l^{2}\right)}\\ \;\mathrm{if}\;\left\{K>\frac{3}{4}l^{2}\right\}\cap \left\{\alpha \leq \frac{2\gamma -1}{1+\gamma }+\frac{3\gamma l^{2}}{2(1+\gamma )(4K-3l^{2})}\right\}\\ (w,\theta )=({\bar{w}}_{\theta ,2},{\bar{\theta }}_{2})\Pi_{\theta ,3}^{m}=2(1+\alpha ){\left(a-c\right)}^{2}\cdot \frac{F_{1}(\tau )+L_{1}(\tau )\sqrt{\Delta_{1}}}{{\left(F_{2}(\tau )+L_{2}(\tau )\sqrt{\Delta_{1}}\right)}^{2}}\\ \;\mathrm{if}\;\left\{\left\{K>\frac{3}{4}l^{2}\right\}\cap \left\{\alpha >\frac{2\gamma -1}{1+\gamma }+\frac{3\gamma l^{2}}{2(1+\gamma )(4K-3l^{2})}\right\}\right\}\cup \\ \;\left\{\left\{K\leq \frac{3}{4}l^{2}\right\}\cap \left\{\left\{\gamma <\bar{\gamma }\right\}\cup \left\{\left\{\gamma \geq \bar{\gamma }\right\}\cap \left\{\alpha >{\bar{\alpha }}_{3}\right\}\right\}\right\}\right\}\\ (w,\theta )=(w_{3}^{*},0)\Pi_{\theta ,3}^{m}=\frac{K(1+\alpha ){\left(a-c\right)}^{2}}{4(1+\alpha +\alpha \gamma )(2K-l^{2})}\\ \;\mathrm{if}\;\left\{K\leq \frac{3}{4}l^{2}\right\}\cap \left\{\left\{\gamma \geq \bar{\gamma }\right\}\cap \left\{0<\alpha \leq {\bar{\alpha }}_{3}\right\}\right\} \end{array}\right..$$ (b.1.7)

By combining (b.1.3), (b.1.5), and (b.1.7), the possible decisions of the manufacturer are $\left(w_{\theta ,1}^{*},\theta_{1}^{*}\right)$, $\left({\bar{w}}_{\theta ,1},{\bar{\theta }}_{1}\right)$ and $\left(w_{\theta ,2}^{*},\theta_{2}^{*}\right)$, according to the different values of *β* for the CS-E contract. Similarly, the possible decisions of the manufacturer are $\left(w_{\theta ,3}^{*},\theta_{3}^{*}\right)$, $\left({\bar{w}}_{\theta ,2},{\bar{\theta }}_{2}\right)$ and $\left(w_{3}^{*},0\right)$, according to the different values of *α*. We summarized these strategies as the manufacturer's local strategy with parameter *β*,*α*. We compared the manufacturer's local optimal profit of *α*,*β* based on *α*≥*β*. Then the manufacturer's global optimal strategy and the corresponding feasible region are shown in Table 2.

### B.2 The retailer's performance for a CS-E contract

The retailer's profit and utility with a CS-E contract that correspond to the manufacturer's optimal strategy (w*,θ*) is presented below:

$$\left\{ \begin{array}{l} U_{\theta ,1}^{r}=\frac{4K(1-\beta ){\left(a-c\right)}^{2}(4K-3l^{2})}{{\left(16K-9l^{2}\right)}^{2}}\Pi_{\theta ,1}^{r}=\frac{2K{\left(a-c\right)}^{2}}{16K-9l^{2}}[\frac{2(4K-3l^{2})}{16K-9l^{2}}-\frac{1-\beta }{1-\beta -\beta \gamma }]\\ \;if\;(w^{*},\theta^{*})=(w_{\theta ,1}^{*},\theta_{1}^{*})\\ U_{\theta ,1}^{r}=\Pi_{\theta ,1}^{r}=2\gamma (1-\beta ){\left(a-c\right)}^{2}\cdot \frac{F_{1}(\tau )+L_{1}(\tau )\sqrt{\Delta_{1}}}{{\left(F_{2}(\tau )+L_{2}(\tau )\sqrt{\Delta_{1}}\right)}^{2}}\tau =\beta \\ \;if\;(w^{*},\theta^{*})=({\bar{w}}_{\theta ,1},{\bar{\theta }}_{1})\\ U_{\theta ,2}^{r}=\Pi_{\theta ,2}^{r}=\frac{K\gamma {\left(a-c\right)}^{2}}{2(1+\gamma )(2K-l^{2})}\\ \;if\;(w^{*},\theta^{*})=(w_{\theta ,2}^{*},\theta_{2}^{*})\\ U_{\theta ,3}^{r}=\frac{4K(1+\alpha ){\left(a-c\right)}^{2}(4K-3l^{2})}{{\left(16K-9l^{2}\right)}^{2}}\Pi_{\theta ,3}^{r}=\frac{2K{\left(a-c\right)}^{2}}{16K-9l^{2}}[\frac{2(4K-3l^{2})}{16K-9l^{2}}-\frac{1+\alpha }{1+\alpha +\alpha \gamma }]\\ \;if\;(w^{*},\theta^{*})=(w_{\theta ,3}^{*},\theta_{3}^{*})\\ U_{\theta ,3}^{r}=\Pi_{\theta ,3}^{r}=2\gamma (1+\alpha ){\left(a-c\right)}^{2}\cdot \frac{F_{1}(\tau )+L_{1}(\tau )\sqrt{\Delta_{1}}}{{\left(F_{2}(\tau )+L_{2}(\tau )\sqrt{\Delta_{1}}\right)}^{2}}\tau =-\alpha \\ \;if\;(w^{*},\theta^{*})=({\bar{w}}_{\theta ,2},{\bar{\theta }}_{2})\\ U_{\theta ,3}^{r}=\frac{K(1+\alpha ){\left(a-c\right)}^{2}}{8(2K-l^{2})}\Pi_{\theta ,3}^{r}=\frac{K{\left(a-c\right)}^{2}(1+\alpha +3\alpha \gamma )}{8(2K-l^{2})(1+\alpha +\alpha \gamma )}\\ \;if\;(w^{*},\theta^{*})=(w_{3}^{*},0) \end{array}\right.$$ (b.2.1)

The retailer's sales effort with a CS-E contract that corresponds to the manufacturer's optimal strategy (w*,θ*), is presented below:

$$\left\{ \begin{array}{l} e_{\theta ,1}^{r}=\frac{6l(a-c)}{16K-9l^{2}}\\ \;if\;(w^{*},\theta^{*})=(w_{\theta ,1}^{*},\theta_{1}^{*})\\ e_{\theta ,1}^{r}=l(1-\beta -\beta \gamma +4\gamma )(a-c)\cdot \frac{8K\gamma +l^{2}(1-\beta -\beta \gamma +3\gamma )+\sqrt{\Delta_{1}}}{\left[(8K+l^{2}(1-\beta -\beta \gamma -3\gamma )]\sqrt{\Delta_{1}}-H(\tau \right)}\tau =\beta \\ \;if\;(w^{*},\theta^{*})=({\bar{w}}_{\theta ,1},{\bar{\theta }}_{1})\\ e_{\theta ,2}^{r}=\frac{l(a-c)}{2K-l^{2}}\\ \;if\;(w^{*},\theta^{*})=(w_{\theta ,2}^{*},\theta_{2}^{*})\\ e_{\theta ,3}^{r}=\frac{6l(a-c)}{16K-9l^{2}}\\ \;if\;(w^{*},\theta^{*})=(w_{\theta ,3}^{*},\theta_{3}^{*})\\ e_{\theta ,3}^{r}=l(1+\alpha +\alpha \gamma +4\gamma )(a-c)\cdot \frac{(8K+3l^{2})\gamma +l^{2}(1+\alpha +\alpha \gamma )+\sqrt{\Delta_{1}}}{\left[(8K-3l^{2})\gamma +l^{2}(1+\alpha +\alpha \gamma )]\sqrt{\Delta_{1}}-H(\tau \right)}\tau =-\alpha \\ \;if\;(w^{*},\theta^{*})=({\bar{w}}_{\theta ,2},{\bar{\theta }}_{2})\\ e_{\theta ,3}^{r}=\frac{l(a-c)}{2(2K-l^{2})}\\ \;if\;(w^{*},\theta^{*})=(w_{3}^{*},0) \end{array}\right.$$ (b.2.2)

where *H*(*τ*) = (1+*γ*)^2^*l*^4^*τ*^2^+2(1+*γ*)(4*Kγ*(4*K*−*l*^2^)−*l*^4^(1+3*γ*))*τ*−8(1+2*γ*)(4*K*−*l*^2^)*Kγ*+(1+3*γ*)^2^*l*^4^

## Appendix C

### C.1 A comparison of the retailer's utility and profit for the two contracts

According to the retailer's utility and profit presented in (a.2.1) and (b.2.1), we can derive the following results using algebraic operation.

For the parameter *β*,

$\left\{ \begin{array}{l} U_{\theta ,1}^{r}<U_{1}^{r}\;\Pi_{\theta ,1}^{r}<\Pi_{1}^{r}\;if\;\left\{0<\beta \leq \frac{1-2\gamma }{1+\gamma }-\frac{3\gamma l^{2}}{2(1+\gamma )(4K-3l^{2})}\right\}\cap \left\{K>\frac{3}{4}l^{2}\right\}\\ U_{\theta ,1}^{r}<U_{1}^{r}\;\Pi_{\theta ,1}^{r}<\Pi_{1}^{r}\;if\;\left\{\beta =0\right\}\cap \left\{\frac{1}{2}l^{2}<K\leq \frac{3}{4}l^{2}\right\}\\ U_{\theta ,1}^{r}>U_{1}^{r}\;\Pi_{\theta ,1}^{r}>\Pi_{1}^{r}\;if\;\left\{\frac{1-2\gamma }{1+\gamma }\leq \beta <\frac{1}{1+\gamma }\right\}\cap \left\{K>\frac{1}{2}l^{2}\right\} \end{array}\right.$; thus, $\left\{ \begin{array}{l} U_{\theta ,1}^{r}<U_{1}^{r}\;\mathrm{if}\;0<\beta <{\bar{\beta }}_{r,1}\\ U_{\theta ,1}^{r}\geq U_{1}^{r}\;\mathrm{if}\;{\bar{\beta }}_{r,1}\leq \beta <\frac{1-2\gamma }{1+\gamma } \end{array}\right.$ and $\left\{ \begin{array}{l} \Pi_{\theta ,1}^{r}<\Pi_{1}^{r}\;\mathrm{if}\;0<\beta <{\bar{\beta }}_{r,2}\\ \Pi_{\theta ,1}^{r}\geq \Pi_{1}^{r}\;\mathrm{if}\;{\bar{\beta }}_{r,2}\leq \beta <\frac{1-2\gamma }{1+\gamma } \end{array}\right.$ in the range $\left\{0<\beta <\frac{1-2\gamma }{1+\gamma }\right\}\cap \left\{K>\frac{1}{2}l^{2}\right\}$, where ${\bar{\beta }}_{r,1}$ is the only real root of the equation $U_{\theta ,1}^{r}({\bar{w}}_{\theta ,1},{\bar{\theta }}_{1})=U_{1}^{r}(w_{1}^{*})$, while ${\bar{\beta }}_{r,2}$ is the only real root of the equation $\Pi_{\theta ,1}^{r}({\bar{w}}_{\theta ,1},{\bar{\theta }}_{1})=\Pi_{1}^{r}(w_{1}^{*})$, and obviously, ${\bar{\beta }}_{r,1}<{\bar{\beta }}_{r,2}<\frac{1-2\gamma }{1+\gamma }$ according to $\Pi_{1}^{r}(w_{1}^{*})>U_{1}^{r}(w_{1}^{*})$.

Similarly, using parameter *α*, then $\left\{ \begin{array}{l} \Pi_{\theta ,3}^{r}\geq \Pi_{3}^{r}\;\mathrm{if}\;\left\{\left\{\alpha \geq {\bar{\alpha }}_{r,2}\right\}\cap \left\{K>\frac{3}{4}l^{2}\right\}\right\}\cup \left\{\left\{4K\leq 3l^{2}\right\}\cap \left\{\left\{\gamma <\bar{\gamma }\right\}\cup \left\{\left\{\gamma \geq \bar{\gamma }\right\}\cap \left\{\alpha >{\bar{\alpha }}_{3}\right\}\right\}\right\}\right\}\\ \Pi_{\theta ,3}^{r}<\Pi_{3}^{r}\;\mathrm{otherwise} \end{array}\right.$, and $\left\{ \begin{array}{l} U_{\theta ,2}^{r}\geq U_{2}^{r}\;\mathrm{if}\;\left\{\left\{\alpha \geq {\bar{\alpha }}_{r,1}\right\}\cap \left\{K>\frac{3}{4}l^{2}\right\}\right\}\cup \left\{\left\{4K\leq 3l^{2}\right\}\cap \left\{\left\{\gamma <\bar{\gamma }\right\}\cup \left\{\left\{\gamma \geq \bar{\gamma }\right\}\cap \left\{\alpha >{\bar{\alpha }}_{3}\right\}\right\}\right\}\right\}\\ U_{\theta ,2}^{r}<U_{2}^{r}\;\mathrm{otherwise} \end{array}\right.$, where ${\bar{\alpha }}_{r,1}$, ${\bar{\alpha }}_{r,2}$ is the only real root of the equation $U_{\theta ,3}^{r}(w_{\theta ,1}^{*},\theta_{1}^{*})=U_{3}^{r}({\tilde{w}}_{2})$ and $\Pi_{\theta ,3}^{r}(w_{\theta ,3}^{*},\theta_{3}^{*})=\Pi_{3}^{r}({\tilde{w}}_{2})$, respectively, and obviously, $\frac{2\gamma -1}{1+\gamma }<{\bar{\alpha }}_{r,2}<{\bar{\alpha }}_{r,1}$.

According to the manufacturer's optimal strategies, which are provided in Tables 1 and 2, we can obtain Proposition 7.

### C.2 A comparison of the channel's total profit with two contracts

Combining the decisions of both the manufacturer and the retailer, the total profit of the supply chain under the two contracts is presented in (c.2.1) and (c.2.2).

1. The channel's total profit under the wholesale price contract is shown below:
  $$\left\{ \begin{array}{l} \Pi_{1}^{c}=\frac{3K{\left(a-c\right)}^{2}}{8(2K-l^{2})}\\ \;\mathrm{if}\;w^{*}=w_{1}^{*}\\ \Pi_{1}^{c}=\frac{2K\gamma (1+\gamma )(1-\beta ){\left(a-c\right)}^{2}}{(2K-l^{2}){\left(1-\beta -\beta \gamma +2\gamma \right)}^{2}}\\ \;\mathrm{if}\;w^{*}={\tilde{w}}_{1}\\ \Pi_{2}^{c}=\frac{K{\left(a-c\right)}^{2}}{2(2K-l^{2})}\\ \;\mathrm{if}\;w^{*}=w_{2}^{*}\\ \Pi_{3}^{c}=\frac{3K{\left(a-c\right)}^{2}}{8(2K-l^{2})}\\ \;\mathrm{if}\;w^{*}=w_{3}^{*}\\ \Pi_{3}^{c}=\frac{2K\gamma (1+\gamma )(1+\alpha ){\left(a-c\right)}^{2}}{(2K-l^{2}){\left(1+\alpha +\alpha \gamma +2\gamma \right)}^{2}}\\ \;\mathrm{if}\;w^{*}={\tilde{w}}_{2} \end{array}\right.$$ (c.2.1)
2. The channel's total profit under the CS-E contract is shown below:
  $$\left\{ \begin{array}{l} \Pi_{\theta ,1}^{c}=\frac{6K{\left(a-c\right)}^{2}(8K-5l^{2})}{{\left(16K-9l^{2}\right)}^{2}}\\ \;\mathrm{if}\;(w^{*},\theta^{*})=(w_{\theta ,1}^{*},\theta_{1}^{*})\\ \Pi_{\theta ,1}^{c}=2(1+\gamma )(1-\beta ){\left(a-c\right)}^{2}\cdot \frac{F_{1}(\tau )+L_{1}(\tau )\sqrt{\Delta_{1}}}{{\left(F_{2}(\tau )+L_{2}(\tau )\sqrt{\Delta_{1}}\right)}^{2}}\tau =\beta \\ \;\mathrm{if}\;(w^{*},\theta^{*})=({\bar{w}}_{\theta ,1},{\bar{\theta }}_{1})\\ \Pi_{\theta ,2}^{c}=\frac{K{\left(a-c\right)}^{2}}{2(2K-l^{2})}\\ \;\mathrm{if}\;(w^{*},\theta^{*})=(w_{\theta ,2}^{*},\theta_{2}^{*})\\ \Pi_{\theta ,3}^{c}=\frac{6K{\left(a-c\right)}^{2}(8K-5l^{2})}{{\left(16K-9l^{2}\right)}^{2}}\\ \;\mathrm{if}\;(w^{*},\theta^{*})=(w_{\theta ,3}^{*},\theta_{3}^{*})\\ \Pi_{\theta ,3}^{c}=2(1+\gamma )(1+\alpha ){\left(a-c\right)}^{2}\cdot \frac{F_{1}(\tau )+L_{1}(\tau )\sqrt{\Delta_{2}}}{{\left(F_{2}(\tau )+L_{2}(\tau )\sqrt{\Delta_{2}}\right)}^{2}}\tau =-\alpha \\ \;\mathrm{if}\;(w^{*},\theta^{*})=({\bar{w}}_{\theta ,2},{\bar{\theta }}_{2})\\ \Pi_{\theta ,3}^{c}=\frac{3K{\left(a-c\right)}^{2}}{8(2K-l^{2})}\\ \;\mathrm{if}\;(w^{*},\theta^{*})=(w_{3}^{*},0) \end{array}\right.$$ (c.2.2)

It is not difficult to determine that $\Pi_{\theta ,1}^{c}\leq \Pi_{1}^{c}$ ($\Pi_{\theta ,1}^{c}>\Pi_{1}^{c}$) if $\beta \leq {\bar{\beta }}_{c}$ ($\beta >{\bar{\beta }}_{c}$) in the interval $K>\frac{1}{2}l^{2}$, where ${\bar{\beta }}_{c}$ is the only real root of the equation $\Pi_{\theta ,1}^{c}({\bar{w}}_{\theta ,1},{\bar{\theta }}_{1})=\Pi_{1}^{c}(w_{1}^{*})$ and ${\bar{\beta }}_{c}<\frac{1-2\gamma }{1+\gamma }$. Similarly, $\left\{ \begin{array}{l} U_{\theta ,3}^{r}<U_{3}^{r}\;\mathrm{if}\;0<\alpha <{\bar{\alpha }}_{c}\\ U_{\theta ,3}^{r}\geq U_{3}^{r}\;\mathrm{if}\;\alpha \geq {\bar{\alpha }}_{c} \end{array}\right.$ for the parameter *α* in the interval $K>\frac{3}{4}l^{2}$ and $U_{\theta ,3}^{r}\geq U_{3}^{r}$ if $\frac{1}{2}l^{2}<K\leq \frac{3}{4}l^{2}$.

Moreover, it is known that $\left\{{\bar{\beta }}_{r,2}|\beta <\frac{1-2\gamma }{1+\gamma }\right\}=\arg(U_{\theta ,1}^{r}({\bar{w}}_{\theta ,1}^{},{\bar{\theta }}_{1})=U_{1}^{r}(w_{1}^{*}))$ and $U_{1}^{r}(w_{1}^{*})=\frac{K(1-\beta ){\left(a-c\right)}^{2}}{8(2K-l^{2})}$, $\Pi_{1}^{c}(w_{1}^{*})=\frac{3K{\left(a-c\right)}^{2}}{8(2K-l^{2})}$; meanwhile, $\Pi_{\theta ,1}^{c}({\bar{w}}_{\theta ,1},{\bar{\theta }}_{1})=\frac{1+\gamma }{\gamma }\cdot \Pi_{\theta ,1}^{r}({\bar{w}}_{\theta ,1},{\bar{\theta }}_{1})=\frac{1+\gamma }{\gamma }\cdot U_{\theta ,1}^{r}({\bar{w}}_{\theta ,1},{\bar{\theta }}_{1})$. Then, $\left\{{\bar{\beta }}_{r,2}|\beta <\frac{1-2\gamma }{1+\gamma }\right\}=\arg(U_{\theta ,1}^{r}({\bar{w}}_{\theta ,1}^{},{\bar{\theta }}_{1})=\frac{K{\left(a-c\right)}^{2}}{8(2K-l^{2})}\cdot (1-\beta ))$ and $\left\{{\bar{\beta }}_{c}|\beta <\frac{1-2\gamma }{1+\gamma }\right\}=\arg(U_{\theta ,1}^{r}({\bar{w}}_{\theta ,1}^{},{\bar{\theta }}_{1})=\frac{K{\left(a-c\right)}^{2}}{8(2K-l^{2})}\cdot \frac{3\gamma }{1+\gamma })$. Obviously, $\frac{3\gamma }{1+\gamma }-(1-\beta )<0$ is in the range $0<\beta <\frac{1-2\gamma }{1+\gamma }$ and $\frac{\partial U_{\theta ,1}^{r}({\bar{w}}_{\theta ,1}^{},{\bar{\theta }}_{1})}{\partial \beta }>0$. Then, ${\bar{\beta }}_{c}<{\bar{\beta }}_{r,2}$ can be inferred, and we can prove ${\bar{\alpha }}_{c}<{\bar{\alpha }}_{r,1}$ in a similar manner.

The analysis above leads to the conclusions in Propositions 8–9.
